# Efficacy and safety of traditional Chinese medicine for intracranial hemorrhage by promoting blood circulation and removing blood stasis: A systematic review and meta-analysis of randomized controlled trials

**DOI:** 10.3389/fphar.2022.942657

**Published:** 2022-09-28

**Authors:** Wenjian Lin, Jingjing Hou, Tianxiong Han, Li Zheng, Huazheng Liang, Xiaoyu Zhou

**Affiliations:** ^1^ Department of Neurology, Shanghai Tenth People’s Hospital, Tongji University School of Medicine, Shanghai, China; ^2^ Tongji University School of Medicine, Shanghai, China; ^3^ Department of Traditional Chinese Medicine, Shanghai Tenth People’s Hospital, Tongji University School of Medicine, Shanghai, China; ^4^ School of Pharmacy, University of Nottingham, Nottingham, United Kingdom; ^5^ Clinical Research Center for Anesthesiology and Perioperative Medicine, Shanghai Fourth People’s Hospital, Tongji University School of Medicine, Shanghai, China; ^6^ Translational Research Institute of Brain and Brain-Like Intelligence, Shanghai Fourth People’s Hospital, Tongji University School of Medicine, Shanghai, China

**Keywords:** intracranial hemorrhage, traditional Chinese medicine, blood stasis, systematic review, meta-analysis

## Abstract

**Background:** Although blood-activating Chinese medicine (BACM) has been reported as adjuvant therapy for intracranial hemorrhage (ICH) in China, high-quality evidence is still lacking. Our study aimed to collect the latest high-quality randomized controlled trials (RCTs) and to evaluate the efficacy and safety of BACM for ICH.

**Methods:** RCTs published between January 2015 and March 2022 were searched in databases, including China National Knowledge Infrastructure (CNKI), China Science and Technology Journal Database (VIP), Sino-Med, Wanfang, PubMed, Web of Science, Cochrane Library, and Embase without language restrictions. Eligible RCTs were included and both primary (clinical efficacy evidenced by decreased neurological deficit scores) and secondary outcomes (increased Barthel index, decreased NIHSS, hematoma volume, the volume of cerebral edema, the incidence of side effects, and mortality) were analyzed. The quality of included RCTs was assessed using the Cochrane risk of bias tool. In the meta-analysis, the pooled results were analyzed using Review Manager 5.3 and STATA14.0. Finally, The GRADEpro GDT software (Guideline Development Tool) was used to summarize the results. Sensitivity and subgroup analyses were conducted based on the follow-up time.

**Results:** Fifteen RCTs, involving 1,579 participants, were included for analysis in our study. The pooled outcomes indicated that BACM combined with western medicine treatment (WMT) was superior to WMT alone for patients with ICH, demonstrated by the improvements in efficacy (RR = 1.22 (95% CI, [1.13 to 1.32], *p* < 0.001), neurological functions (MD_NIHSS_ = −2.75, 95% CI [−3.74 to −1.76], *p* < 0.001), and activities of daily living (MD_Barthel index_ = 5.95, 95% CI [3.92 to 7.98], *p* < 0.001), as well as decreased cerebral hematoma, cerebral edema (MD cerebral hematoma = −2.94, 95% CI [−3.50 to −2.37, *p* < 0.001 and MD_cerebral edema_ = −2.66, 95% CI [−2.95 to −2.37], *p* < 0.001), side effects and mortality (RR = 0.84 (95% CI [0.60 to 1.19], *p* = 0.330 and RR = 0.51 (95% CI, [0.16 to 1.65], *p* = 0.260). In addition, *Conioselinum anthriscoides “Chuanxiong”* [Apiaceae], *Camellia reticulata Lindl.* [Theaceae], and *Bupleurum sibiricum var. jeholense* (Nakai) C.D.Chu [Apiaceae]) were the most frequently used herbs in the treatment of ICH. Recently, there was a trend toward the extensive use of another two herbs, including *Rheum palmatum L.* [Polygonaceae], *Astragalus mongholicus Bunge* [Fabaceae]) for ICH.

**Conclusion:** BACM combined with WMT seems to be superior to WMT alone for patients with ICH. Further high-quality RCTs are warranted to confirm the efficacy and safety of BACM.

## Introduction

Intracranial hemorrhage (ICH) is one of the most devastating types of stroke with an incidence of 24.6 per 100,000 individuals, imposing a heavy burden on the global health care system (Staykov et al., 2010; Poon et al., 2014). Recent evidence has shown that surgical treatment improves neurologic functions in neither short nor long terms after ICH (Hostettler et al., 2019). In addition, no pharmacological agents have been proven to improve the clinical outcome of patients with ICH (Hemphill et al., 2015; Ni et al., 2020).

According to the Traditional Chinese Medicine (TCM) theory, ICH is the result of specific injuries that accumulate in individuals, such as emotional irritability, improper diet, overwork, stagnation of Qi and Blood, endogenous Phlegm, imbalance of Yin and Yang, and other incentives. Therefore, ICH is related to “Wind,” “Extra heat,” “Phlegm,” “Blood stasis,” “Qi” and “Deficiency” (Liu and Guo, 2009; Zhang and Xing, 2019). Compared with other syndromes, “Blood stasis” is one of the most common syndromes in ICH (Yang et al., 2004; Zhang and Zhang, 2021) because “Wind,” “Extra heat,” “Phlegm,” “Qi” and “Deficiency” eventually evolve into “Blood stasis.” Therefore, “Blood stasis” is the leading cause of ICH and occurs throughout the whole process of ICH (Yang and Yan, 2014). In the “Blood Evidence,” Tang Rongchuan proposed that blood that leaves a blood vessel leads to “Blood stasis.” Modern Chinese medicine research suggests that “Blood stasis” obstructs the flow of new blood and impacts the recovery of hemorrhagic stroke patients (Zeng et al., 2022). TCM theory describes blood activation as promoting the body’s “Qi” and blood and restoring the body to a vigorous state. The purpose of activating blood circulation and removing blood stasis is to restore the normal blood flow and to improve the patient’s clinical symptoms. Therefore, use of blood-activating Chinese medicine (BACM) by hemorrhagic stroke patients is reasonable. An expert consensus from TCM also pointed out that BACM is the primary choice for ICH patients (Gao 2016). Effective removal of hematoma is essential for functional recovery in patients with ICH (Li et al., 2022).

Inflammatory response, cytotoxicity, and oxidative stress occur after ICH (Zhu et al., 2019). Research has shown close association of “Blood stasis” with blood viscosity, vascular endothelial permeability, and endothelial relaxation factors (Song, 2000). In TCM, *Achyranthes bidentata Blume* [Amaranthaceae], *Camellia reticulata Lindl.* [Theaceae], *Conioselinum anthriscoides ‘Chuanxiong’* [Apiaceae], and *Salvia miltiorrhiza Bunge* [Lamiaceae] are termed blood-activating Chinese herbs (BACH), which are effective in harmonizing the blood and removing blood stasis (Liu et al., 2019). For example, *Salvia miltiorrhiza Bunge* [Lamiaceae] is widely used, and it has the potential to prevent cerebral edema (Zhang et al., 2003). *Conioselinum anthriscoides ‘Chuanxiong’* [Apiaceae] can reduce the release of endothelin and inhibit cytotoxicity (Sun et al., 2008). *Camellia reticulata Lindl.* [Theaceae] acts as an antioxidant through its potent ability to scavenge free radicals (Pang et al., 2020). Furthermore, emerging evidence has shown that BACM increases concentrations of nerve growth factor and brain-derived neurotrophic factor in the plasma, reduces levels of homocysteine, tumor necrosis factor α, matrix metalloprotein 9, interleukin-6 (IL-6), and hypersensitive-c-reactive-protein in the serum (Wang et al., 2016a; Xi et al., 2018; Lin et al., 2022).

Recently, with the use of classical prescriptions, self-made prescriptions, or Chinese patent medicines, an array of randomized controlled trials (RCTs) on intracranial hemorrhage have been reported, focusing on the essence of activating blood circulation and removing blood stasis. Therefore, we aimed to analyze RCTs on BACM combined with western medicine treatments (WMT) such as Mannitol Injection, Sodium nitroprusside injection, and Symptomatic treatment prescribed for ICH patients to provide more reliable clinical evidence on BACM in managing ICH. Finally, reporting of this review is in strict accordance with the Preferred Reporting Items for Systematic Review and Meta-Analyses (PRISMA) 2020 statement (Page et al., 2021).

## Methods

### Protocol registration

The Systematic Review and Meta-Analysis was registered in PROSPERO (Registration Number: CRD42022326159).

### Information sources

RCTs published between January 2015 and March 2022 were searched in the following databases and registries: PubMed, Web of Science, Cochrane Library, Embase, Chinese Biomedical Literature Service System (SinoMed), China National Knowledge Infrastructure (CNKI), Chinese Scientific Journals Database (VIP), WanFang database, ClinicalTrials.gov, and Chinese Clinical Trial Register (ChiCTR).

### Searching strategy

The following keywords were used in different combinations to search literatures published between January 2015 and March 2022: “Intracranial Hemorrhage,” “Cerebral Hemorrhage,” “SHU XUE,” “HUA YU,” “HUO XUE,” “blood activating agents,” “stasis removing agents,” and “activating blood.” The detailed searching strategies for all databases were presented in the Supplementary Text.

### Inclusion criteria

#### Types of studies

All included articles were RCTs, including dissertations and journal articles.

#### Types of participants

According to the guidelines, we included participants of any age or gender with a primary clinical diagnosis of intracranial hemorrhage (Chinese Society of Neurology and Chinese Stroke Society, 2019).

#### Types of interventions

Intervention groups were treated with TCM alone or combined with WMT. In contrast, control groups were treated with one or more WMT, without the use of TCM herbs or other therapies. WMT in control groups included drugs for dehydration and intracranial pressure reduction, maintenance of water and electrolyte balance, control of blood pressure, blood sugar and lipids, and symptomatic treatment.

#### Types of outcomes

The primary outcomes was clinical efficacy, calculated with the following equation (The fourth session of the National Cerebrovascular Conference, 1996): effective clinical rate (%) (number of patients with recovery + number of patients with significant improvement + number of patients with improvement)/total number of patients × 100%. The reduction of neurological deficit score (NDS) was determined as an efficacy criterion. Recovery was defined as the reduction of 91–100% in NDS. Significant improvement was the reduction of 46–90% in NDS, and improvement was the decrease of 18–45% in NDS, while ineffectiveness was the drop of NDS to less than 17%.

Secondary outcomes included the reduction of the National Institute of Health Stroke Scale (NIHSS) and increased activities of daily living (Barthel Index). In addition, the volume of hematoma, the volume of cerebral edema, the incidence of side effects, and mortality were assessed as secondary outcomes.

### Exclusion criteria

(1) Reviews, meta-analysis, basic medical research, or animal research;
(2) Secondary cerebral hemorrhage, including trauma, tumor, intracranial vascular malformation, subarachnoid hemorrhage, *etc*;
(3) Hemorrhagic transformation after ischemic stroke;
(4) Incomplete information or uncalculated outcomes;
(5) Both treatment groups and control groups were treated with traditional Chinese medicine combined with other therapies;
(6) No control group;
(7) BACM sequential therapy.

### Study selection

**FIGURE 1:**
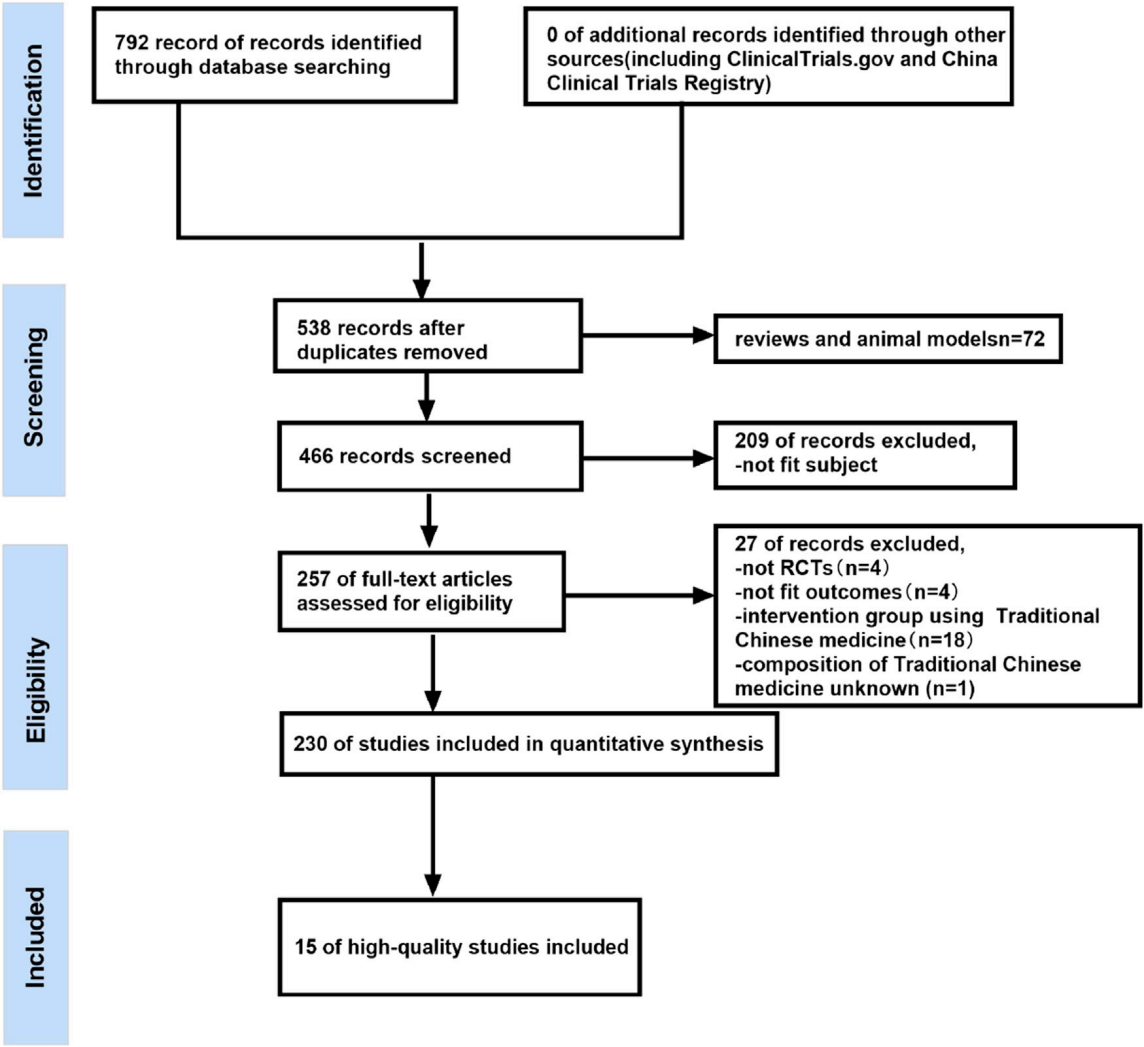
Flow chart of the article selection process.

Two authors independently searched the databases for eligible RCTs based on the inclusion and exclusion criteria. After removing duplicate records and animal experiments, the records retrieved from all databases were imported into NoteExpress 3.5.0 and Endnote 20.3 and screened based on the title and abstract. Finally, some studies were excluded due to their failure to meet the inclusion criteria. Figure 1 showed details of the study selection process.

### Data extraction

Two researchers independently used a pre-prepared extraction form to extract data from the included articles and cross-checked the data. Disagreement between researchers was resolved through consultation with a third reviewer until a consensus was reached. Data extracted from included studies were: *1*) Name of the first author; *2*) Year of publishing; *3*) Country of study; *4*) Mean age and mean volume of hematoma of patients; *5*) The number of patients and sex ratio (male to female); *6*) Course of treatment; *7*) Time of onset; *8*) Interventions in the BACM and the control groups; *9*) Results of articles (If studies adopted different follow-up time points for patients, we would separately extract results).

### Quality assessment

Selected articles were independently reviewed by two researchers based on seven aspects (including generation of random sequences, allocation concealment, blinding of subjects, investigators and outcome assessors, incomplete outcome reporting, selective outcome reporting, and other biases) using the Cochrane Risk of Bias Tool (Higgins et al., 2011). Articles with “low risk” in each domain were included for further analysis, whereas those with “unclear risk” or “high risk” in ≥3 domains were excluded (Chung et al., 2013). Conflicts were resolved by a third researcher or discussion until a consensus was reached.

### Data analysis

Review Manager 5.3 was used for meta-analysis. The effect size was calculated using an odds ratio (OR) with 95% CI for dichotomous data. Continuous variables were expressed as the weighted mean difference (MD). Standardized mean difference (SMD) was considered when the measurement units differed. All variables were demonstrated with their effect sizes and their 95% confidence intervals (CIs). In addition, the fixed-effect model was used when heterogeneity was evaluated by Chi-square (Chi) test and I^2^ statistic (if *p* ≥ 0.05, or I^2^ ≤ 50%). Otherwise, the random-effect model was used. Furthermore, sensitivity analysis was conducted to evaluate the stability of outcomes and the overall efficacy of BACM combined with WMT for ICH treatment. Based on the patient’s follow-up time, subgroup analyses were conducted. Publication bias was evaluated through STATA14.0 using Begg’s test, Egger’s test, and funnel plot.

### Assessment of evidence

The GRADE pro-GDT software (Guideline Development Tool) was used to evaluate the outcomes (Deng et al., 2019; Duan et al., 2021).

## Results

### Study selection

The detailed literature search and study selection process were shown in Figure 1. A total of 792 records were found using various searching strategies, inclusion and exclusion criteria. Among them, 254 records were duplicates, and 72 records were reviews or models. Furthermore, 209 articles were excluded because of inappropriate subjects. Then, 257 studies were manually screened according to the exclusion and inclusion criteria. Of these, four studies were not RCTs, four studies were unsuitable for outcomes analysis, one study did not state the composition of traditional Chinese medicine, and the control group in 18 studies also used BACM. Finally, A total of 15 high-quality studies were included after two researchers independently assessed 230 articles using the Cochrane Risk of Bias Tool.

### Study characteristics

Characteristics of the included RCTs were provided in Table 1 and Supplementary Tables S1–S15. A total of 15 RCTs (Li, 2015a; Li, 2015b; Li, 2015c; Li et al., 2016a; Kong et al., 2015; Yuan et al., 2015; Sun, 2017; Zhang et al., 2017; Gui, 2018; Xu, 2018; Zhang, 2018; Gao et al., 2020; Chen et al., 2021; Lv et al., 2021; Qiu et al., 2021) involving 1,579 participants were included in this analysis, with 784 participants in the intervention group and 795 participants in the control group. The majority of patients had an onset to the presentation time of less than 24 h, with a maximum of 72 h, and the duration of treatment is mainly 2 weeks to 8 weeks, with a maximum of 90 days. The types of drugs in the intervention group were herbal prescriptions, classical decoctions, and Chinese patent medicines, including capsules and injections. The sample size of included studies ranged from 20 to 108, and all included studies were conducted in China with WMT used in the control group, including surgical treatment, blood pressure control, glycemic control, and reduction of intracranial pressure. BACM was used as an intervention strategy. Seven RCTs employed the effective clinical rate as an outcome measure, and 14 RCTs reported NIHSS as their outcome measures. In addition, hematoma volume in 10 articles and cerebral edema volume in eight articles were considered outcome measures, respectively. Additionally, two articles used the Barthel Index, the incidence of side effects, and mortality as outcome measures.

**TABLE 1:** Characteristics of the included RCTs.

Included study	Country	Male/Female (Case)		The course of treatment (day)	Time of onset (hours)	Groups		Included main outcomes
		Intervention	Control			Intervention	Control	
Zhang kaichuang 2018	China	12/8	11/9	14	≤24 h	Self-made Suihai Huayu Decoction+WMT	WMT (Mannitol Injection + Sodium nitroprusside injection + Spearhead viper hemocoagulase injection)	①
Chen Qingwei 2021	China	30/17	34/13	28	<24 h	Tongqiao Huoxue Decoction+WMT	WMT (Fasudil hydrochloride injection + Hemagglutinin injection + Mannitol injection + Dexmedetomidine hydrochloride injection + Naloxone hydrochloride injection)	①②③⑥
Gao Jiying 2020	China	29/24	31/22	14	3–72 h	Huoxue Ditan Decoction+Hyperbaric oxygen therapy+WMT	Hyperbaric oxygen therapy+WMT (Routine therapy of reducing intracranial pressure + Hyperbaric oxygen therapy + Symptomatic treatment)	①⑤
Xu Quantao 2018	China	31/21	33/19	14	14–67 h	Self-made Bushen Ditan Huayu Decoction+WMT	WMT (Routine therapy of reducing intracranial pressure + Symptomatic treatment)	⑤
Gui Zhong 2018	China	11/9	10/10	14	—	Zhuling Siwu Decoction+WMT	WMT (Mannitol injection + Antihypertensive therapy + Symptomatic treatment)	①④
Zhang Fang 2017	China	37/17	31/23	14	≤24 h	Huoxue Sanyu Xingnao Decoction+Surgical treatment+WMT	Surgical treatment+WMT (Symptomatic treatment)	①②⑦
Li Zongwu 2015	China	18/12	17/13	14	<15 h	Naomai Xinshen Capsules+WMT	WMT (Routine therapy of reducing intracranial pressure + Symptomatic treatment)	④⑤
Yuan Lixin 2015	China	71/43	70/44	21	<72 h	Huoxue Huayu Decoction+WMT	WMT (Routine therapy of reducing intracranial pressure + Symptomatic treatment)	③
Li Lili 2015	China	25/22	27/20	14	<96 h	Huoxue Ditan Decoction+Surgical treatment+WMT	Surgical treatment+WMT (Routine therapy of reducing intracranial pressure+ Symptomatic treatment)	①②⑤
Kong Wei 2015	China	15/10	17/8	56	<24 h	Self-made Xiaozhong Huayu Decoction+WMT	WMT (Routine therapy of reducing intracranial pressure + Symptomatic treatment)	④
Lv Yue 2021	China	32/23	35/20	56	<6 h	Sanyu Tongluo Decoction+WMT	WMT (Mannitol injection + Nimodipine tablets + Levoamlodipine + Edaravone injection)	①②④⑤
Li Sujuan 2015	China	34/18	32/20	15	—	Huoxue Huayu Decoction+WMT	WMT (Routine therapy of reducing intracranial pressure + Symptomatic treatment)	①④
Sun Chuanhe 2017	China	32/19	29/21	12	<24 h	Naoxueshu Oral Liquid+WMT	WMT (Routine therapy of reducing intracranial pressure + Symptomatic treatment)	②
Li Jingya 2016	China	31/71	41/67	90	<72 h	Xingnaojing Injection+Naoxueshu Injection+Huoxue Huayu Decoction+WMT	WMT (Routine therapy of reducing intracranial pressure + Symptomatic treatment)	②④⑥⑦
Qiu You 2021	China	49/25	55/19	28	<24 h	Yiqi Huoxue Huayu Decoction+Surgical treatment+WMT	Surgical treatment+WMT (Routine therapy of reducing intracranial pressure+ Symptomatic treatment)	①②④

WMT, Western medicine treatment; ①, Clinical efficacy; ②, NIHSS; ③, Barthel Index; ④, The volume of hematoma; ⑤, The volume of cerebral edema; ⑥, Side effects; ⑦, Mortality.

### Assessment of risk of bias

**FIGURE 2:**
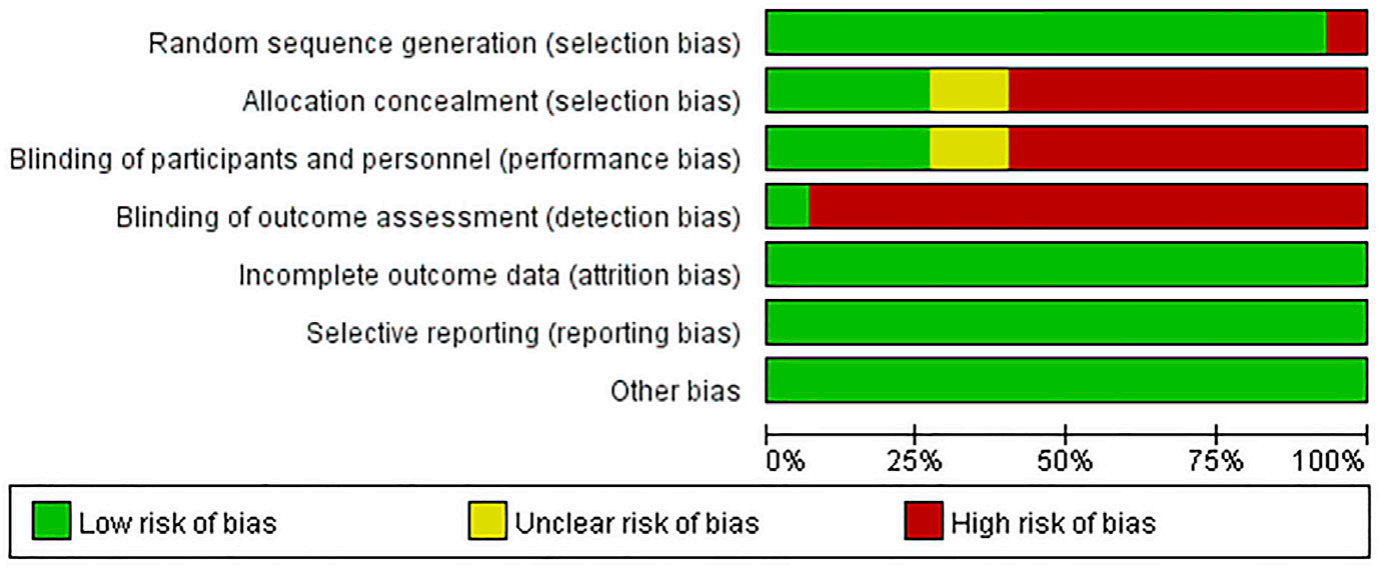
Risk of bias graph for included RCTs.

**FIGURE 3:**
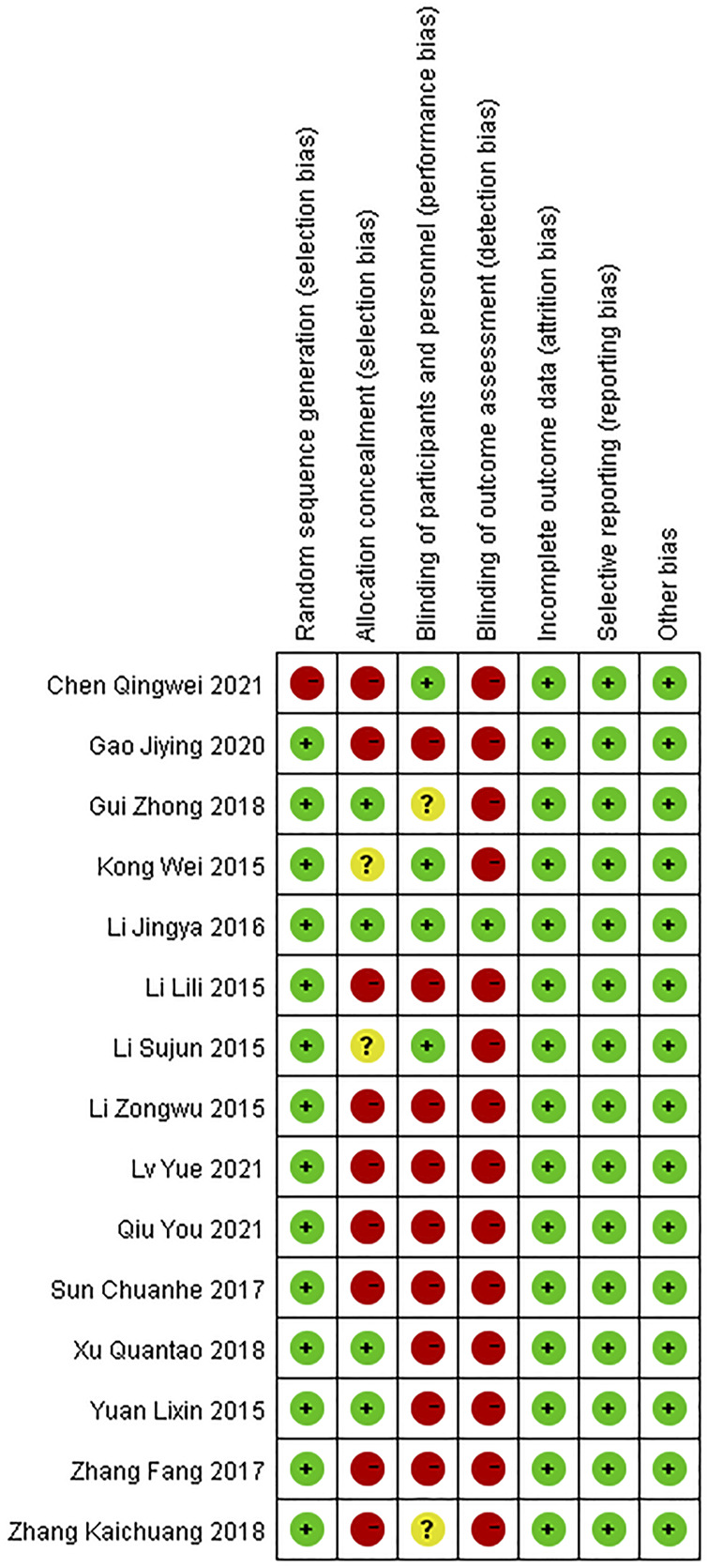
Risk of bias summary for included RCTs.

Results on the assessment of risk of bias of included studies were shown in Figures 2, 3. The Cochrane Risk of Bias Tool was used to assess this risk based on seven domains.

#### Domain 1: risk of bias arising from the randomization process

For the generation of random sequences, 14 trials were ranked “low risk” because the authors used a random number table (Li, 2015a; Li, 2015b; Li, 2015c; Kong et al., 2015; Yuan et al., 2015; Li et al., 2016b; Sun, 2017; Zhang et al., 2017; Gui, 2018; Xu, 2018; Zhang, 2018; Gao et al., 2020; Lv et al., 2021; Qiu et al., 2021); one trial was ranked “high risk” because patients were allocated to the control and treatment groups using the date of birth odd (Chen et al., 2021).

#### Domain 2: risk of bias arising from the allocation concealment

For allocation concealment, four trials were considered“low risk” because they used opaque envelopes to randomize patients (Yuan et al., 2015; Li et al., 2016a; Gui, 2018; Xu, 2018). The allocation concealment was not mentioned in the rest of the trials. Nine trials were assessed as “high risk” because they conducted a random number table to assign patients (Li, 2015a; Li, 2015b; Sun, 2017; Zhang et al., 2017; Zhang, 2018; Gao et al., 2020; Chen et al., 2021; Lv et al., 2021; Qiu et al., 2021). Another two trials were regarded as “unclear risk” because they used a random method to assign patients and did not mention details of concealment (Li, 2015c; Kong et al., 2015).

#### Domain 3: risk of bias arising from blinding of participants and researchers

Four articles were regarded as “low risk” because these studies explicitly stated how participants and personnel were blinded (Kong et al., 2015; Li, 2015a; Li et al., 2016b; Chen et al., 2021). Two trials were regarded as “unclear risk” because these trials described the personnel who were blinded to the trial, and there was insufficient evidence to confirm participants were also blinded (Gui, 2018; Zhang, 2018). Nine articles were considered “high risk” because the authors did not provide sufficient information on whether personnel or participants were blinded to measures of treatment (Yuan et al., 2015; Li, 2015a; Li, 2015b; Sun, 2017; Zhang et al., 2017; Xu, 2018; Gao et al., 2020; Lv et al., 2021; Qiu et al., 2021).

#### Domain 4: risk of bias in the measurement of outcomes

One trial was considered “low risk” because the trial mentioned blinding of results (Li et al., 2016a). The rest of trials were judged as “high risk” because they did not state whether results were blinded or not.

#### Domain 5: risk of bias due to missing outcome data

Since complete clinical data of all patients were available, all trials were judged as “low risk of bias” in this domain.

#### Domain 6: risk of bias in the selection of the reported results

Outcomes in these trials were displayed without selection bias. We judged all trials as “low risk of bias.”

#### Domain 7: risk of bias from other ways

No other risk of bias was found in these 15 trials.

### The use of the blood-activating Chinese herbs

Among the 230 RCTs, 15 herbs were used most frequently, including *Conioselinum anthriscoides ‘Chuanxiong’* [Apiaceae] (53.28%), *Camellia reticulata Lindl.* [Theaceae] (49.34%), *Bupleurum sibiricum var. jeholense* (Nakai) C.D.Chu [Apiaceae] (48.47%), *Prunus persica* (L.) *Batsch* [Rosaceae] (47.60%), *Rheum palmatum L.* [Polygonaceae] (39.30%), Earthworm (38.86%), *Astragalus mongholicus Bunge* [Fabaceae] (34.50), *Salvia miltiorrhiza Bunge* [Lamiaceae] (34.50), *Angelica sinensis* (Oliv.) Diels [Apiaceae] (27.07), *Achyranthes bidentata Blume* [Amaranthaceae] (26.64%), *Panax notoginseng* (Burkill) F.H.Chen [Araliaceae] (26.64%), Leech (23.14%), *Acorus calamus L.* [Acoraceae] (17.90%), *Rehmannia glutinosa* (Gaertn.) DC. [Orobanchaceae] (14.85%), and *Gastrodia elata Blume* [Orchidaceae] (14.41%) in the forms of decoctions, injections, and oral granules/capsules/liquid. Among these 15 herbs, *Rheum palmatum L.* [Polygonaceae], *Rehmannia glutinosa* (Gaertn.) DC. [Orobanchaceae], *Acorus calamus L.* [Acoraceae], and *Gastrodia elata Blume* [Orchidaceae] were considered BACH to be used for patients with ICH in recent years. In addition, among the 15 RCTs, 15 Chinese herbs were commonly used, including *Rheum palmatum L.* [Polygonaceae] (80%), *Conioselinum anthriscoides ‘Chuanxiong’* [Apiaceae] (80%), *Camellia reticulata Lindl.* [Theaceae] (60%), *Astragalus mongholicus Bunge* [Fabaceae] (53.3%), *Prunus persica* (L.) *Batsch* [Rosaceae] (46.67%), *Panax notoginseng* (Burkill) F.H.Chen [Araliaceae] (40%), *Acorus calamus L.* [Acoraceae] (40%), *Angelica sinensis* (Oliv.) Diels [Apiaceae] (33.33%), Earthworm (33.33%), *Bupleurum sibiricum var. jeholense* (Nakai) C.D.Chu [Apiaceae] (33.33%), *Salvia miltiorrhiza Bunge* [Lamiaceae] (33.33%), Leech (33.33%), *Gastrodia elata Blume* [Orchidaceae] (33.33%), *Alisma plantago-aquatica L.* [Alismataceae] (26.67%), *Achyranthes bidentata Blume* [Amaranthaceae] (26.67%), *Wolfiporia cocos* (F.A. Wolf) Ryvarden & Gilb (26.67%). Among15 included trials, the dosage of 15 BACH in treatment of ICH and the pharmacological mechanisms of these Chinese herbs were shown in Table 2.

**TABLE 2:** Among 15 included trials, the dosage of 15 BACH in treatment of ICH and pharmacological mechanisms of these Chinese herbs.

Included study	The decoction of name	The decoction of composition and dosage	The decoction of instructions	The most commonly used Chinese medicine in 15 RCTs	Frequency of using herbs in 15 RCTs	Frequency of use in 230 RCTs	Mechanism of single herb for ICH	Reference	The pharmacological mechanisms of the most frequently used herbs in 15 RCTs
Zhang kaichuang 2018	Self-made Suihai Huayu Decoction	*Astragalus mongholicus Bunge* [Fabaceae] 30 g, Fresh Oyster 15 g, *Typha angustifolia L.* [Typhaceae] 15 g, *Angelica sinensis* (Oliv.) Diels10 g, *Rheum palmatum L.* [Polygonaceae] 10 g, *Spatholobus suberectus Dunn* [Fabaceae] 10 g, *Polygonatum sibiricum Redouté* [Asparagaceae] 10 g, *Wolfiporia cocos* (F.A. Wolf) Ryvarden & Gilb 10 g, *Glycyrrhiza uralensis Fisch. ex DC.* [Fabaceae] 10 g, *Alisma plantago-aquatica L.* [Alismataceae] 10 g, *Panax notoginseng* (Burkill) F.H.Chen [Araliaceae] 10 g, *Prunus persica* (L.) *Batsch* [Rosaceae] 10 g, *Conioselinum anthriscoides “Chuanxiong”* [Apiaceae] 10 g, *Rehmannia glutinosa* (Gaertn.) DC. [Orobanchaceae] 10 g, Earthworm 10 g, *Panax ginseng C.A.Mey*. [Araliaceae] 5 g, *Achyranthes bidentata Blume* [Amaranthaceae] 5 g, Leech 3 g	Decocting 1 dose a day (about 300 ± 5 ml) and taking it two times daily	Conioselinum anthriscoides “Chuanxiong” [Apiaceae]	12 (80.00%)	122 (53.28%)	**·**↑ the expression levels of ET in serum	Sun et al. (2008)	①, ④, ⑤, ⑥, ⑦, ⑨, ⑩, ⑪, ⑫, ⑬, ⑮
Chen Qingwei 2021	Tongqiao Huoxue Decoction	two *Allium fistulosum L.* [Amaryllidaceae], *Lilium longiflorum Thunb.* [Liliaceae] 1 g, *Ziziphus jujuba Mill.* [Rhamnaceae] 10 g, *Rheum palmatum L.* [Polygonaceae] 10 g, *Bupleurum sibiricum var. jeholense* (Nakai) C.D.Chu [Apiaceae] 15 g, *Camellia reticulata Lindl.* [Theaceae] 10 g, *Prunus persica* (L.) *Batsch* [Rosaceae] 10 g, *Astragalus mongholicus Bunge* [Fabaceae] 50 g, *Conioselinum anthriscoides ‘Chuanxiong’* [Apiaceae] 15 g	After mixing the herbs, using 1,000 ml of boiling water to decoct to 200 ml, and taking it two times daily, each time 100 ml	*Rheum palmatum L.* [Polygonaceae]	12 (80.00%)	90 (39.30%)	**·**↑ the ability to preserve BBB integrity	Wang et al. (2016b)	①, ②, ③, ④, ⑤, ⑦, ⑬
							**·**↑ the expression levels of ZO-1 protein		
							**·**↑ the ability to inhibit the inflammatory response, curb neuronal cell necrosis and apoptosis, and resist oxidative stress	Yuan et al. (2017)	
Gao Jiying 2020	Huoxue Ditan Decoction	*Salvia miltiorrhiza Bunge* [Lamiaceae] 30 g, *Astragalus mongholicus Bunge* [Fabaceae] 30 g, *Ginkgo biloba L.* [Ginkgoaceae] 15 g, *Lycium chinense Mill.* [Solanaceae] 15 g, *Gastrodia elata Blume* [Orchidaceae] 15 g, *Acorus calamus L.* [Acoraceae] 15 g, *Conioselinum anthriscoides ‘Chuanxiong’* [Apiaceae] 10 g, *Alisma plantago-aquatica L.* [Alismataceae] 15 g, *Rheum palmatum L.* [Polygonaceae] 10 g, *Pueraria montana var. lobata* (Willd.) Maesen & S.M.Almeida ex Sanjappa & Predeep [Fabaceae] 10g, *Camellia reticulata Lindl.* [Theaceae] 6 g, *Prunus persica* (L.) *Batsch* [Rosaceae] 10 g, Earthworm 10 g	Decocting 1 dose a day (about 200 ml)and taking two times daily	*Camellia reticulata Lindl.* [Theaceae]	9 (60.00%)	113 (49.34%)	**·**↓ the expression levels of intracellular caspase-3 protein	Ma et al. (2013)	①, ②, ④, ⑤, ⑥, ⑦, ⑧, ⑬, ⑭, ⑯
							**·**↑ the ability to delay cell apoptosis		
							**·**↑ the ability of synaptic plasticity	(Li et al., 2016a)	
							**·**↑ the ability to resist lipid peroxidative damage, resist oxygen free radicals, and improve hemodynamics	Pang et al. (2020)	
Xu Quantao 2018	Self-made Bushen Ditan Huayu Decoction	*Astragalus mongholicus Bunge* [Fabaceae] 30 g, *Lycium chinense Mill.* [Solanaceae], *Reynoutria multiflora* (Thunb.) *Moldenke* [Polygonaceae], *Salvia miltiorrhiza Bunge* [Lamiaceae], *Ginkgo biloba L.* [Ginkgoaceae], *Acorus calamus L.* [Acoraceae] 15 g respectively, Earthworm, *Camellia reticulata Lindl.* [Theaceae], *Bambusa tuldoides Munro* [Poaceae], *Allium fistulosum L.* [Amaryllidaceae], Citrus × aurantium L. [Rutaceae] 10 g respectively	Decocting 1 dose a day and taking it two times daily	*Astragalus mongholicus Bunge* [Fabaceae]	8 (53.33%)	79 (34.50%)	**·**↓the expression levels of NSE in the serum and Notch1 and NF-κB protein in the brain tissue	Xie et al. (2016)	②, ⑥, ⑦, ⑧, ⑬
							**·**↑the ability to stimulate immunity and decrease inflammation	Chen et al. (2017)	
Gui Zhong 2018	Zhuling Siwu Decoction	Polyporus umbellatus (Pers.)Fries 10 g, *Wolfiporia cocos* (F.A. Wolf) Ryvarden & Gilb 10 g, *Alisma plantago-aquatica L.* [Alismataceae] 10 g, Equus asinus L. 10 g, Talcum 10 g, *Rehmannia glutinosa* (Gaertn.) DC. [Orobanchaceae] 12 g, *Angelica sinensis* (Oliv.) Diels 10 g, *Paeonia lactiflora Pall.* [Paeoniaceae] 12 g, *Conioselinum anthriscoides “Chuanxiong”* [Apiaceae] 8 g	After mixing the herbs, use 300 ml of water to decoct to 150 ml, and take it 1 one time daily	*Prunus persica* (L.) *Batsch* [Rosaceae]	7 (46.67%)	109 (47.60%)	-		①, ⑨, ⑮, ⑯
Zhang Fang 2017	Huoxue Sanyu Xingnao Decoction	*Astragalus mongholicus Bunge* [Fabaceae] 60 g, *Angelica sinensis* (Oliv.) Diels 15 g, *Conioselinum anthriscoides “Chuanxiong”* [Apiaceae] 15 g, *Prunus persica* (L.) *Batsch* [Rosaceae] 12 g, *Camellia reticulata Lindl.* [Theaceae] 20 g, *Bupleurum sibiricum var. jeholense* (Nakai) C.D.Chu [Apiaceae] 20 g, *Gastrodia elata Blume* [Orchidaceae] 15 g, *Uncaria rhynchophylla (Miq.) Miq.* [Rubiaceae] 15 g, Earthworm 10 g, *Rheum palmatum L.* [Polygonaceae] 12 g, Citrus × aurantium L. [Rutaceae] 10 g	Decocting 1 dose a day and taking two times after adding powder of *Panax notoginseng* (Burkill) F.H.Chen [Araliaceae] 3 g	*Panax notoginseng (Burkill) F.H.Chen* [Araliaceae]	6 (40.00%)	61 (26.64%)	**·**↓ the expression levels of Bax and Caspase-3	Qiu et al. (2017)	①, ②, ③, ④, ⑤, ⑥, ⑦, ⑨, ⑬, ⑭
							**·**↑ the expression levels of Bcl-2		
							**·**↑ the ability to reduce neuronal apoptosis		
Li Zongwu 2015	Naomai Xinshen Capsules	*Rheum palmatum L.* [Polygonaceae], *Prunus persica* (L.) *Batsch* [Rosaceae], *Camellia reticulata Lindl.* [Theaceae], *Conioselinum anthriscoides “Chuanxiong”* [Apiaceae], *Panax notoginseng* (Burkill) F.H.Chen [Araliaceae], *Salvia miltiorrhiza Bunge* [Lamiaceae]	Taking three times, each time 0.99 g	*Acorus calamus L.* [Acoraceae]	6 (40.00%)	41 (17.90%)	—		①, ②, ④, ⑤, ⑧, ⑩
Yuan Lixin 2015	Huoxue Huayu Decoction	Decoction 1: *Panax notoginseng* (Burkill) F.H.Chen [Araliaceae] 10 g, *Curcuma zedoaria (Christm.) Roscoe* [Zingiberaceae] 10 g, *Typha angustifolia L.* [Typhaceae] 9 g, *Rheum palmatum L.* [Polygonaceae] 3 g, *Gardenia jasminoides J.Ellis* [Rubiaceae] 10 g, *Scutellaria baicalensis Georgi* [Lamiaceae] 10 g, *Wolfiporia cocos* (F.A. Wolf) Ryvarden & Gilb 20 g, *Trichosanthes kirilowii Maxim.* [Cucurbitaceae] 20 g	Huoxue Huayu Decoction conducted granules without decoction, taking two times daily, 150 ml each time, Xingnaojing injection 20ml, one time daily, and Naoxueshu Oral Liquid 10ml, three times daily	*Gastrodia elata Blume* [Orchidaceae]	5 (33.33%)	33 (14.41)	**·**↑the ability to dilate cerebral blood vessels, antagonize hypoxia and reduce cerebrovascular resistance	He et al. (2012)	Decoction 1:⑤, ⑮
		Decoction 2: *Panax notoginseng* (Burkill) F.H.Chen [Araliaceae] 10 g, *Curcuma zedoaria (Christm.) Roscoe* [Zingiberaceae] 10 g, *Typha angustifolia L.* [Typhaceae] 9 g, *Gastrodia elata Blume* [Orchidaceae] 12 g, *Uncaria rhynchophylla (Miq.) Miq.* [Rubiaceae] 15 g, *Bupleurum sibiricum var. jeholense* (Nakai) C.D.Chu [Apiaceae] 12 g, *Wolfiporia cocos* (F.A. Wolf) Ryvarden & Gilb 20 g, Haliotidis Concha 30 g							Decoction 2:③, ⑭, ⑮
		Decoction 3: *Panax notoginseng* (Burkill) F.H.Chen [Araliaceae] 10 g, *Curcuma zedoaria (Christm.) Roscoe* [Zingiberaceae] 10 g, *Typha angustifolia L.* [Typhaceae] 9 g, *Trichosanthes kirilowii Maxim.* [Cucurbitaceae] 30 g, Pinellia ternata (Thunb.) Makino [Araceae] 10 g, *Wolfiporia cocos* (F.A. Wolf) Ryvarden & Gilb 20 g, *Rheum palmatum L.* [Polygonaceae] 3 g, three decoction according to dialectical classification					**·**↑the levels of cerebral blood flow		Decoction 3:⑤, ⑮
Li Lili 2015	Huoxue Ditan Decoction	*Salvia miltiorrhiza Bunge* [Lamiaceae] 20 g, *Astragalus mongholicus Bunge* [Fabaceae] 50 g, *Lycium chinense Mill.* [Solanaceae] 15 g, *Ginkgo biloba L.* [Ginkgoaceae] 15 g, *Reynoutria multiflora* (Thunb.) *Moldenke* [Polygonaceae] 15 g, *Gastrodia elata Blume* [Orchidaceae] 15 g, *Acorus calamus L.* [Acoraceae] 15 g, *Conioselinum anthriscoides “Chuanxiong”* [Apiaceae] 10 g, *Alisma plantago-aquatica L.* [Alismataceae] 15 g, *Bambusa tuldoides Munro* [Poaceae] 10 g, *Pueraria montana var. lobata* (Willd.) Maesen & S.M.Almeida ex Sanjappa & Predeep [Fabaceae] 10 g, Earthworm 10 g, *Rheum palmatum L.* [Polygonaceae] 10 g	Decocting 1 dose a day and taking it two times daily	Earthworm	5 (33.33%)	89 (38.86%)	-		①, ⑤, ⑥, ⑦, ⑧, ⑬, ⑭, ⑯
Kong Wei 2015	Self-made Xiaozhong Huayu Decoction	*Panax notoginseng* (Burkill) F.H.Chen [Araliaceae] 5 g, Blood scorpion 3 g, *Conioselinum anthriscoides “Chuanxiong”* [Apiaceae] 10 g, *Rheum palmatum L.* [Polygonaceae] 10 g, Completed Scorpio 15 g, *Curcuma aromatica Salisb.* [Zingiberaceae] 15 g, Arisaema Cum Bile 15 g, *Acorus calamus L.* [Acoraceae] 15 g, Leech 10 g, *Camellia reticulata Lindl.* [Theaceae] 10 g, *Prunus persica* (L.) *Batsch* [Rosaceae] 10 g, Mother of pearl 10 g	Decocting 1 dose a day (about 150 ml)and taking two times	*Bupleurum sibiricum var. jeholense* (Nakai) C.D.Chu [Apiaceae]	5 (33.33%)	111 (48.47%)	-		①, ②, ④, ⑤, ⑩, ⑬, ⑫
Lv Yue 2021	Sanyu Tongluo Decoction	*Panax notoginseng* (Burkill) F.H.Chen [Araliaceae] 12 g, *Conioselinum anthriscoides “Chuanxiong”* [Apiaceae] 10 g, *Bupleurum sibiricum var. jeholense* (Nakai) C.D.Chu [Apiaceae] 12 g, *Camellia reticulata Lindl.* [Theaceae] 10 g, *Rheum palmatum L.* [Polygonaceae] 12 g, *Typha angustifolia L.* [Typhaceae] 10 g, Buffalo horn; 30 g, *Dryobalanops aromatica C.F.Gaertn.* [Dipterocarpaceae] 0.1 g	Decocting 1 dose a day (about 400 ml)and taking two times daily, 200 ml each time	*Salvia miltiorrhiza Bunge* [Lamiaceae]	5 (33.33%)	79 (34.50%)	**·**↑ the ability to reduce the degree of cerebral edema	Zhang et al. (2003)	①, ②, ③, ⑤, ⑩
Li Sujuan 2015	Huoxue Huayu Decoction	*Angelica sinensis* (Oliv.) Diels 10 g, *Biancaea sappan* (L.) *Tod.* [Fabaceae] 5 g, *Bupleurum sibiricum var. jeholense* (Nakai) C.D.Chu [Apiaceae] 10 g, *Astragalus mongholicus Bunge* [Fabaceae] 30 g, *Achyranthes bidentata Blume* [Amaranthaceae] 10 g, Leech 5 g, Antelope Horn 5 g, *Conioselinum anthriscoides “Chuanxiong”* [Apiaceae] 10 g, *Camellia reticulata Lindl.* [Theaceae] 10 g, *Prunus persica* (L.) *Batsch* [Rosaceae] 6 g	Decocting 1 dose a day (about 300 ml), once every 4 h for severe cases, once every 6 h for mild cases, 60–100 ml each time	*Angelica sinensis* (Oliv.) Diels [Apiaceae]	5 (33.33%)	62 (27.07%)	**-**		①, ②, ③, ④, ⑦, ⑨, ⑪, ⑫, ⑬
Sun Chuanhe 2017	Naoxueshu Oral Liquid	*Astragalus mongholicus Bunge* [Fabaceae], leech, *Acorus calamus L.* [Acoraceae], *Achyranthes bidentata Blume* [Amaranthaceae], *Paeonia × suffruticosa Andrews* [Paeoniaceae], *Rheum palmatum L.* [Polygonaceae], *Conioselinum anthriscoides “Chuanxiong”* [Apiaceae]	Taking three times, each time 10 ml	Leech	5 (33.33%)	53 (23.14%)	·↑ the ability to activate the endogenous fibrinolytic system	Wu et al. (2007)	①, ⑤, ⑦, ⑪, ⑫, ⑬
							**·**↑ the ability to reduce inflammatory, dissolving blood clots and improve microcirculation	(Liu et al., 2017; Tang et al., 2020)	
Li Jingya 2016	Xingnaojing Injection+Naoxues huInjection+Huoxue Huayu Decoction	**Decoction 1:** *Panax notoginseng* (Burkill) F.H.Chen [Araliaceae] 10 g, *Curcuma zedoaria (Christm.) Roscoe* [Zingiberaceae] 10 g, *Typha angustifolia L.* [Typhaceae] 9 g, *Rheum palmatum L.* [Polygonaceae] 3 g, *Gardenia jasminoides J.Ellis* [Rubiaceae] 10 g, *Scutellaria baicalensis Georgi* [Lamiaceae] 10 g, *Wolfiporia cocos* (F.A. Wolf) Ryvarden & Gilb 20 g, *Trichosanthes kirilowii Maxim.* [Cucurbitaceae] 20 g	The methods of using Huoxue Huayu Decoction unknown, Xingnaojing injection 20ml, one time daily, and Naoxueshu Oral Liquid 10ml, three times daily	*Wolfiporia cocos* (F.A. Wolf) Ryvarden & Gilb	4 (26.67%)	+	-		Decoction 1: ⑤, ⑮
		**Decoction 2:** *Panax notoginseng* (Burkill) F.H.Chen [Araliaceae] 10 g, *Curcuma zedoaria (Christm.) Roscoe* [Zingiberaceae] 10 g, *Typha angustifolia L.* [Typhaceae] 9 g, *Gastrodia elata Blume* [Orchidaceae] 12 g, *Uncaria rhynchophylla (Miq.) Miq.* [Rubiaceae]15 g, *Bupleurum sibiricum var. jeholense* (Nakai) C.D.Chu [Apiaceae] 12 g, *Wolfiporia cocos* (F.A. Wolf) Ryvarden & Gilb 20 g, Haliotidis Concha 30 g							Decoction 2: ③, ⑭, ⑮
		**Decoction 3:** *Panax notoginseng* (Burkill) F.H.Chen [Araliaceae] 10 g, *Curcuma zedoaria (Christm.) Roscoe* [Zingiberaceae] 10 g, *Typha angustifolia L.* [Typhaceae] 9 g, *Trichosanthes kirilowii Maxim.* [Cucurbitaceae] 30 g, Pinellia ternata (Thunb.) Makino [Araceae] 10 g, *Wolfiporia cocos* (F.A. Wolf) Ryvarden & Gilb 20 g, *Rheum palmatum L.* [Polygonaceae ] 3 g, three decoction according to dialectical classification							Decoction 3: ⑤, ⑮
Qiu You 2021	Huayu Decoction	*Salvia miltiorrhiza Bunge* [Lamiaceae] 30 g, *Acorus calamus L.* [Acoraceae], *Bambusa textilis McClure* [Poaceae] 20 g respectively, *Angelica sinensis* (Oliv.) Diels, *Rehmannia glutinosa* (Gaertn.) DC. [Orobanchaceae], Leech, *Rheum palmatum L.* [Polygonaceae], *Camellia reticulata Lindl.* [Theaceae], *Achyranthes bidentata Blume* [Amaranthaceae] 15 g respectively, *Conioselinum anthriscoides ‘Chuanxiong’* [Apiaceae], *Curcuma aromatica Salisb.* [Zingiberaceae], Arisaema Cum Bile 12 g respectively, Sodium Sulfate 6 g	Decocting 1 dose a day (about 200 ml) and taking two times daily	*Alisma plantago-aquatica L.* [Alismataceae]	4 (26.67%)	+	-		①, ②, ⑤, ⑧, ⑨, ⑫, ⑬
				Achyranthes bidentata Blume [Amaranthaceae]	4 (26.67%)	61 (26.64%)	-		
				*Rehmannia glutinosa* (Gaertn.) DC. [Orobanchaceae]	+	34 (14.85%)	·↑ the ability to accelerate coagulation and protect BBB	(Bimpis et al., 2015; Wang et al., 2015)	

①, *Conioselinum anthriscoides ‘Chuanxiong’* [Apiaceae]; ②, *Camellia reticulata Lindl.* [Theaceae]; ③, *Bupleurum sibiricum var. jeholense* (Nakai) C.D.Chu [Apiaceae]; ④, *Prunus persica* (L.) *Batsch* [Rosaceae]; ⑤, *Rheum palmatum L.* [Polygonaceae]; ⑥, Earthworm; ⑦, *Astragalus mongholicus Bunge* [Fabaceae]; ⑧, *Salvia miltiorrhiza Bunge* [Lamiaceae]; ⑨, *Angelica sinensis* (Oliv.) Diels [Apiaceae]; ⑩, *Panax notoginseng* (Burkill) F.H.Chen [Araliaceae]; ⑪, *Achyranthes bidentata Blume* [Amaranthaceae]; ⑫, Leech; ⑬, *Acorus calamus L.* [Acoraceae]; ⑭, *Gastrodia elata Blume* [Orchidaceae]; ⑮, *Wolfiporia cocos* (F.A. Wolf) Ryvarden & Gilb; ⑯, *Alisma plantago-aquatica L.* [Alismataceae]; g, gram; −, No articles were found on the mechanism of single herbs for intracerebral hemorrhage; +, These herbs was not most frequently used in included RCTs.

### Efficacy outcomes

#### Clinical effectiveness rate

**FIGURE 4:**
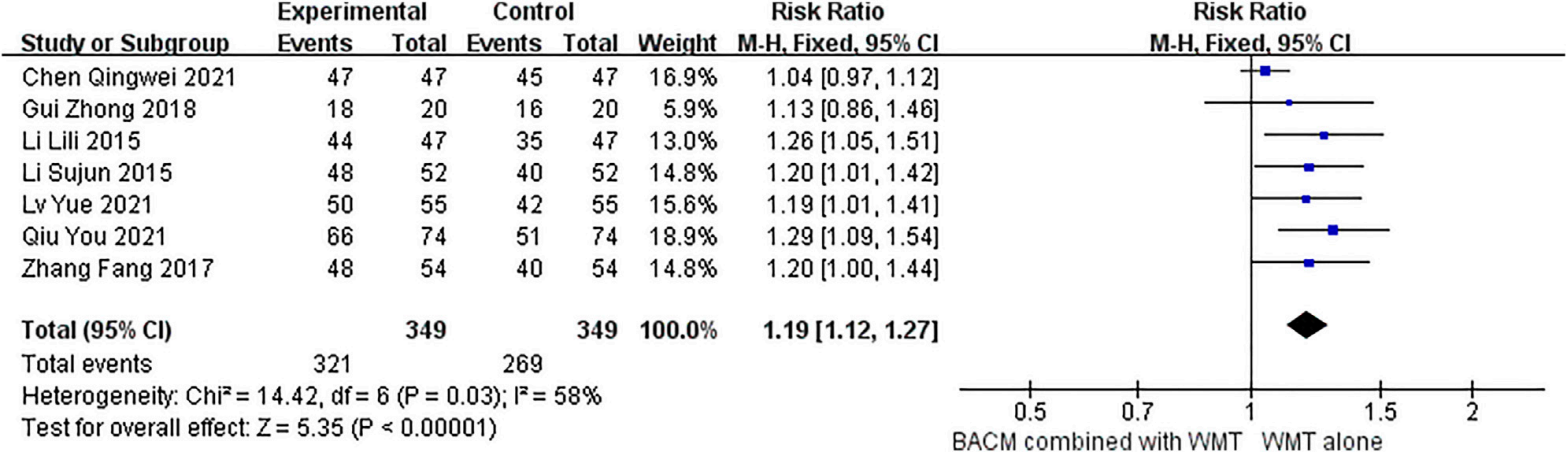
The forest plot demonstrating the clinical efficacy of BACM for ICH.

Seven studies (Zhang et al., 2017; Gui, 2018; Li, 2015a; Li, 2015b; Chen et al., 2021; Lv et al., 2021; Qiu et al., 2021) used the change of NIHSS as the marker of clinical effectiveness. As shown in Figure 4, we evaluated the efficacy of BACM plus WMT with the RR values. The Random-effects model was used due to the presence of strong heterogeneity (I^2^ = 58%; χ^2^ = 14.42; df = 6, *p* = 0.03). The difference was statistically significant, with the pooled RR of 1.19 (95% CI [1.12 to 1.27], *p* < 0.001). We omitted trials one-by-one and found that the RR was 1.22 (95% CI [1.13 to 1.32], *p* < 0.001) with little heterogeneity (I^2^ = 0%, *p* = 0.96) when the study (Chen et al., 2021) was deleted (Supplementary Figure S1). From the forest plot, the CI of this trial (Chen et al., 2021) had a smaller coverage than the other studies. We hypothesized that participants in this study (Chen et al., 2021) had milder clinical symptoms and better outcomes than patients in other studies because participants in Chen’s study (Chen et al., 2021) had the lowest mean NIHSS among the pooled trials. The funnel plot for publication bias (Supplementary Figure S2) did not show significant publication bias among the six RCTs, with Begg’s test (Z = 0.38, *p* = 0.707) and Egger’s test (T = −1.29, *p* = 0.2) showing insignificant difference.

#### NIHSS

**FIGURE 5:**
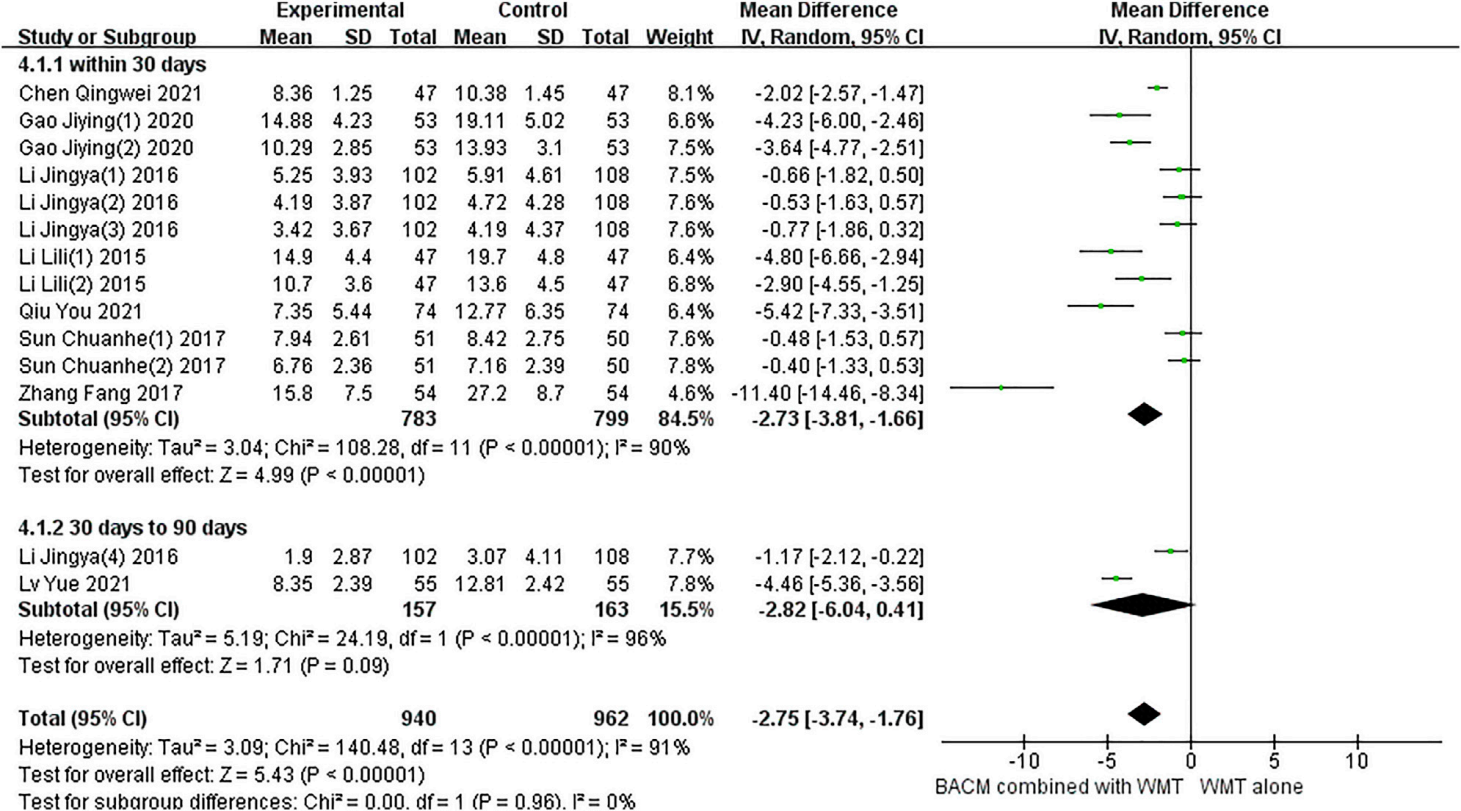
The forest plot demonstrating improvement of NIHSS by BACM for ICH.

Eight RCTs with fourteen results reported the NIHSS in patients with ICH.and a total of 10 studies included multiple time points: day 5 (Sun, 2017), day 7 (Li et al., 2015; Li et al., 2016a; Gao et al., 2020), day 10 (Sun, 2017), day 14 (Gao et al., 2020; Zhang et al., 2017; Li, 2015a; Li et al., 2016a), day 21 (Li et al., 2016b), day 28 (Chen et al., 2021; Qiu et al., 2021), day 56 (Lv et al., 2021), day 90 (Li et al., 2016a). As shown in Figure 5, due to statistically significant heterogeneity (I^2^ = 91%; χ^2^ = 140.48; df = 13, *p* < 0.001), conclusions were obtained using a random-effects model (MD =−2.75, 95% CI [−3.74 to −1.76], *p* < 0.001) and subgroup analysis was performed according to time points. Twelve trials, including participants who received treatment for less than 30 days, were significantly heterogeneous (*p* < 0.001, I^2^ = 90%). The results of pooled twelve RCTs (Sun, 2017; Sun, 2017; Zhang et al., 2017; Li, 2015a; Li, 2015b; Li et al., 2016a; Li et al., 2016b; Li et al., 2016b; Gao et al., 2020; Gao et al., 2020; Chen et al., 2021; Qiu et al., 2021) showed that the difference was statistically significant (MD = −2.73, 95% CI [−3.81 to −1.66], *p* < 0.001). In addition, there was significant heterogeneity between the two RCTs (Li et al., 2016a; Lv et al., 2021) with a duration of 56 and 90 days, respectively (*p* < 0.01, I^2^ = 96%), and the results showed no statistically significant difference (MD = −2.82, 95% CI [−6.04 to 0.41], *p* = 0.09).

The significant heterogeneity in this study may be attributed to the fact that different types of BACM removing blood stasis had different therapeutic effects on patients with NDS. For example, participants in the study of Zhang et al. (Zhang et al., 2017) had more severe NDS, which may explain the presence of greater efficacy observed in this trial than in others. To examine publication bias, funnel plots were shown in Supplementary Figure S3, and the results of Begg’s test (Z = 2.08, *p* = 0.037) and Egger’s test (T = −1.82, *p* = 0.94) indicated that there was no significant publication bias in the 14 RCTs.

#### Barthel index

**FIGURE 6:**
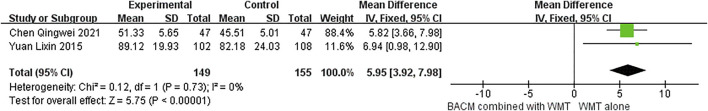
The forest plot of improved Barthel index by BACM for ICH.

The Barthel index was reported in two RCTs (Yuan et al., 2015; Chen et al., 2021). As shown in Figure 6, BACM that promoted blood circulation and removed blood stasis combined with WMT was superior to WMT alone in improving patients’ quality of life (MD = 5.95,95% CI [3.92,7.98], *p* < 0.001). However, the result needs to be interpreted with caution due to the small sample size.

#### Hematoma volume

**FIGURE 7:**
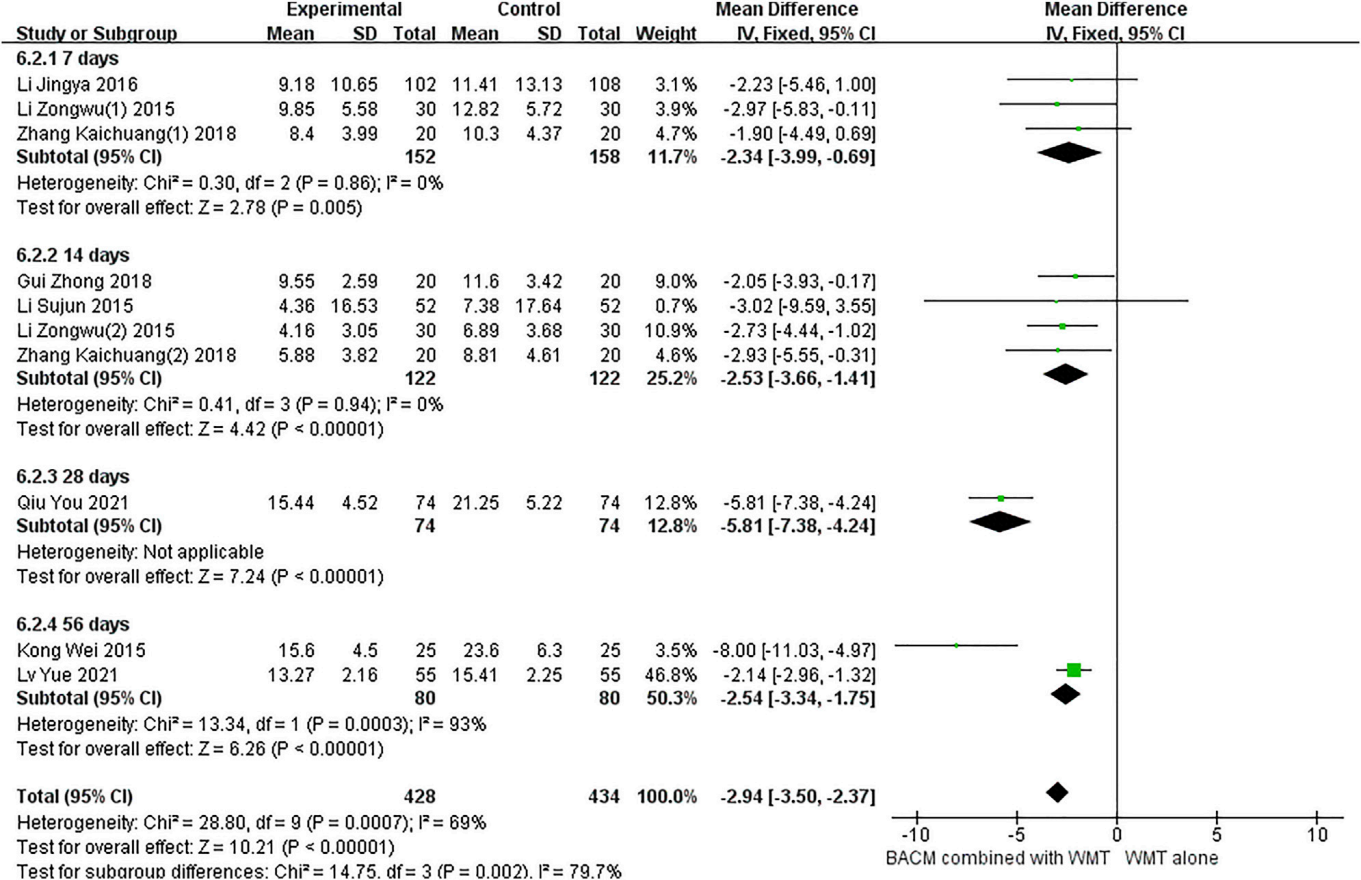
The forest plot of the volume of cerebral hematoma by BACM for ICH.

Of the fifteen trials, 10 (Li et al., 2016a; Li, 2015a; Li, 2015b; Li, 2015c; Zhang, 2018; Zhang, 2018; Gui, 2018; Qiu et al., 2021; Kong et al., 2015; Lv et al., 2021) used hematoma volume as a clinical outcome. As shown in Figure 7, we used a random-effects model (MD = −2.94, 95% CI [−3.50 to −2.37], *p* < 0.001) due to the presence of significant heterogeneity (*p* < 0.001, I^2^ = 69%). Clinical outcomes were measured with the change of hematoma volume after 1 week of onset in three RCTs (Li et al., 2016a; Li, 2015c; Zhang, 2018), 2 weeks in four RCTs (Zhang, 2018; Gui, 2018; Li, 2015a; Li, 2015b), 3 weeks in one RCT (Qiu et al., 2021), and 8 weeks in two RCTs (Kong et al., 2015; Lv et al., 2021). There was no heterogeneity between participants who underwent head CT scans after 1 and 2 weeks, respectively (*p* = 0.86, I^2^ = 0%; *p* = 0.94, I^2^ = 0%), and the results were statistically significant (MD_1 week_ = −2.34, 95% CI [−3.99 to −0.69, *p* = 0. 005; MD_2 week_ = −2.53, 95% CI [−3.66 to −1.41], *p* < 0.001). In addition, there was significant heterogeneity in the group of patients who underwent CT scan after 8 weeks (I^2^ = 93%, *p* = 0.0003). We speculated that the sample size of this study (Kong et al., 2015) was smaller than that of the other study (Lv et al., 2021), leading to an exaggerated absorption of cerebral hematoma in the group of BACM combined with WMT. As shown in Supplementary Figure S4, Begg’s test (Z = 1.25, *p* = 0.210) and Egger’s test (T = −0.93, *p* = 0.379) indicated the absence of significant publication bias in 10 trials.

#### Cerebral edema

**FIGURE 8:**
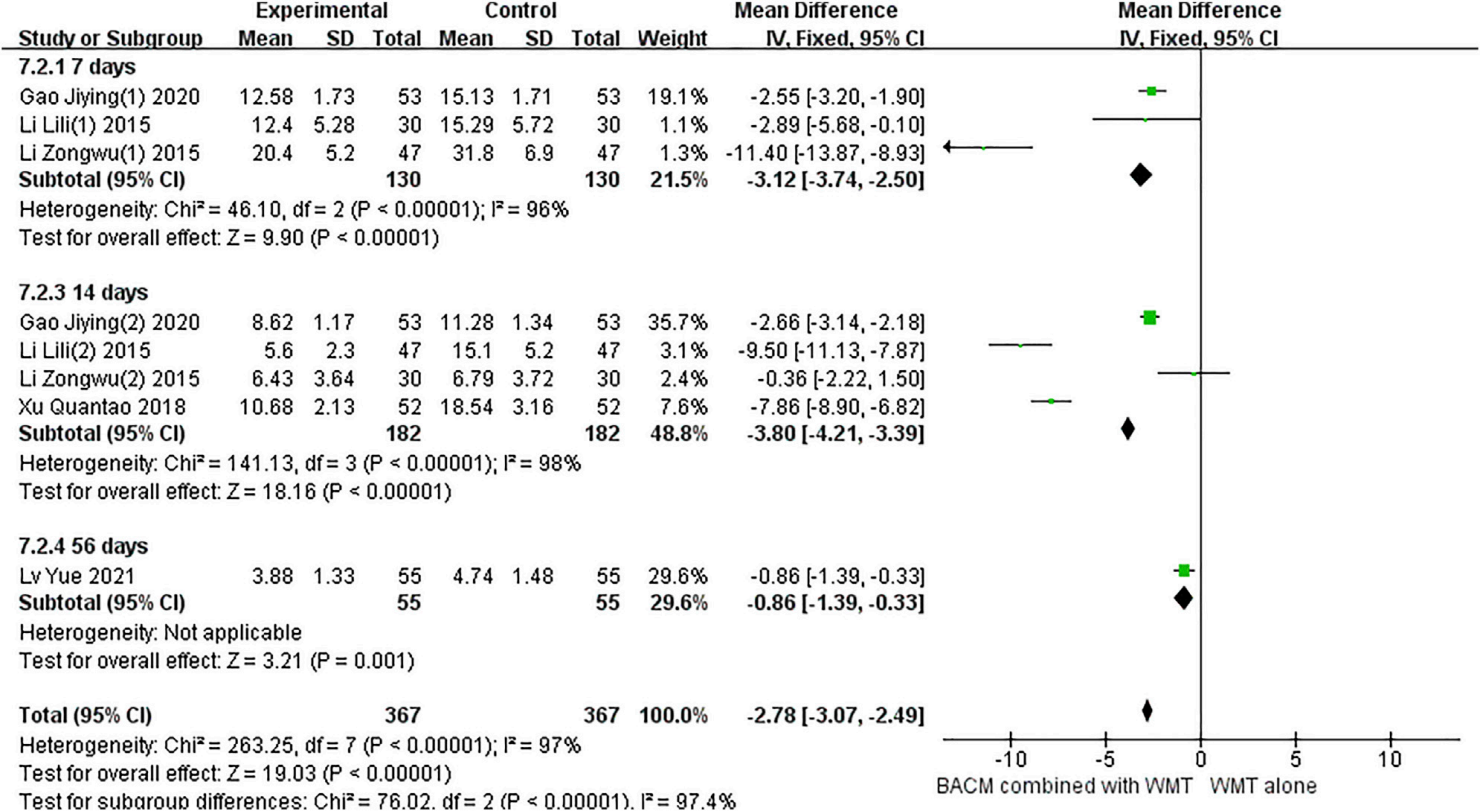
The forest plot of the volume of cerebral edema by BACM for ICH.

Eight trials reported cerebral edema (Xu, 2018; Gao et al., 2020; Li, 2015a; Li, 2015b; Li, 2015c; Li, 2015; Gao et al., 2020; Lv et al., 2021). As shown in Figure 8, the heterogeneity was significant (I^2^ = 97%, *p* < 0.001), therefore, a random-effects model was used. We found that BACM combined with WMT was associated with a reduction in brain edema volume compared with WMT alone (MD = −2.74, 95% CI [−3.01 to −2.48], *p* < 0.001). Three RCTs (Gao et al., 2020; Li, 2015a; Li, 2015b) performed the second head CT scan after 1 week, four RCTs (Xu, 2018; Gao et al., 2020; Li, 2015a; Li, 2015b) performed the second head CT scan after 2 weeks, and one RCT (Lv et al., 2021) performed the second head CT scan after 8 weeks. Subgroup analysis showed that there was significant heterogeneity between the 1-week groups and the 2-week groups. In addition, we found that Li’s study (Li, 2015c) had significant heterogeneity. When this trial (Li, 2015a) was excluded, the heterogeneity was improved (I^2^ = 0, *p* = 0.82), and the difference was statistically significant (MD 1-week group = −2.57, 95%CI [−3.21 to −1.93], *p* < 0.001; MD_total_ = −2.66, 95%CI [−2.95 to −2.37], *p* < 0.00001), as shown in Supplementary Figure S5). The small sample size and small mean edema volume in Li’s trial (Li, 2015a) brought about exaggerated effects and heterogeneity. Because absorption of cerebral edema was faster in Li’s study (Li L., 2015) than in the other two RCTs (Gao et al., 2020; Li, 2015a), the pooled value was larger. The effect of BACM combined with WMT was superior to that of WMT alone in the 1-week group (Gao et al., 2020; Li, 2015a) (MD = −2.57, 95% CI [−3.21 to −1,83], *p* < 0.001). In addition, there was significant heterogeneity in the pooled effect size (MD = −3.80, 95% CI [−4.21 to −3. 39]) for the four RCTs (Xu, 2018; Gao et al., 2020; Li, 2015a; Li, 2015b) at 2 weeks (I^2^ = 98%, *p* < 0.001). Apart from the baseline level of brain edema volume, no other factors contributed to this heterogeneity. To examine publication bias, funnel plots were shown in Supplementary Figure S6, and the results of Begg’s test (Z = 0.30, *p* = 0.764) and Egger’s test (T = −1.95, *p* = 0.109) did not show significant publication bias in seven RCTs.

#### Adverse reactions

**FIGURE 9:**
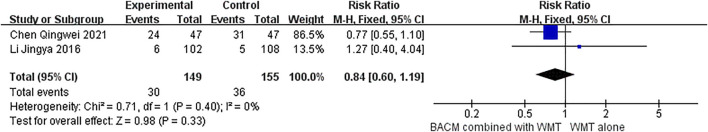
The forest plot of the incidence of side effects due to BACM for ICH.

**FIGURE 10:**
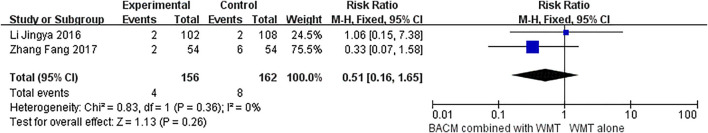
The forest plot of the incidence of mortality due to BACM for ICH.

Two RCTs (Chen et al., 2021; Li et al., 2016a) demonstrated that BACM combined with WMT elicited adverse reactions in patients with ICH during the intervention. Chen et al. (Chen et al., 2021) showed that the BACM (Tongqiao Huoxue decoction) used by patients with ICH had side effects, including hepatic impairment in three patients, renal impairment in three patients, gastrointestinal reaction in nine patients, intracranial infection in four patients, pulmonary infections in four patients, and subcutaneous hemorrhage in one patient. However, some of these adverse reactions might be indirectly related to the intake of BACM. In addition, another study (Li et al., 2016b) reported adverse events in seven patients when BACM (Xingnaojing Injection/Naoxueshu Injection/Huoxue Huayu Decoction) was prescribed to patients with ICH, including pressure ulcer, diarrhea, deep venous thrombosis, allergy to BACM (Xingnaojing), renal function impairment, increased hematoma, and two deaths due to impaired renal function and increased hematoma. In the study by Zhang et al. (Zhang et al., 2017), two deaths were also reported. However, no statistical difference was found in the incidence of side effects and mortality between BACM and control groups (RR = 0.84 (95% CI [0.60 to 1.19], *p* = 0.33 and RR = 0.51 (95% CI, [0.16 to 1.65], *p* = 0.26), with forest plots shown in Figures 9, 10, respectively.

### Sensitivity analysis

Sensitivity analysis was performed on the clinical effectiveness (Zhang et al., 2017; Gui, 2018; Li, 2015a; Li, 2015b; Chen et al., 2021; Lv et al., 2021; Qiu et al., 2021), hematoma volume (Li, 2015a; Li, 2015b; Li, 2015c; Li et al., 2016a; Zhang, 2018; Zhang, 2018; Gui, 2018; Qiu et al., 2021; Kong et al., 2015; Lv et al., 2021), and cerebral edema (Gao et al., 2020; Li, 2015a; Li, 2015b; Li, 2015c; Li, 2015; Gao et al., 2020; Xu, 2018; Lv et al., 2021). To reveal the stability of results, including clinical effectiveness, hematoma volume, and cerebral edema in the meta-analysis, we excluded RCTs one by one. None of these exclusions changed the statistical significance, suggesting that our results were robust. However, there was significant heterogeneity in cerebral hematoma, cerebral edema, and NIHSS.

### Evaluation of quality

The GRADEpro Guideline Development Tool was used to assess the quality of evidence for nine outcomes, including the clinical effectiveness (Li, 2015a; Li, 2015b; Zhang et al., 2017; Gui, 2018; Chen et al., 2021; Lv et al., 2021; Qiu et al., 2021), NIHSS on day 30 (Li, 2015a; Li et al., 2016a; Li et al., 2016b; Li et al., 2016; Sun, 2017; Gao et al., 2021; Li et al., 2021; Sun, 2017; Gao et al., 2020; Zhang et al., 2017; Chen et al., 2021; Qiu et al., 2021), NIHSS from 30 to 90 days (Lv et al., 2021; Li et al., 2016a), Barthel index (Yuan et al., 2015; Chen et al., 2021), hematoma volume on day 7 (Li et al., 2016a; Li, 2015a; Zhang, 2018), hematoma volume on day 14 (Li, 2015a; Li, 2015b; Zhang, 2018; Gui, 2018), hematoma volume on day 56 (Kong et al., 2015; Lv et al., 2021), cerebral edema volume on day 7 (Li, 2015a; Li, 2015b; Gao et al., 2020), the volume of cerebral edema on day 14 (Li, 2015a; Li, 2015b; Xu, 2018; Gao et al., 2020). The levels of evidence ranged from very low, low, moderate, to high. Results were shown in Table 3.

**TABLE 3:** GRADE summary of outcomes for blood-activating Chinese medicine to patients with intracerebral hemorrhage.

Certainty assessment							№ of patients		Effect		Certainty	Importance
No. of studies	Study design	Risk of bias	Inconsistency	Indirectness	Imprecision	Other considerations	TCM combined with WMT	WMT alone	Relative (95% CI)	Absolute (95% CI)		
The clinical effective rate												
6	randomized trials	serious^a^	not serious	not serious	not serious	none	302/274 (110.2%)	224/302 (74.2%)	RR 1.22 (1.13–1.32)	**1**63 more per 1,000 (from 96 more to 237 more)	⊕⊕⊕○ Moderate	
NIHSS - within 30 days												
12	randomized trials	serious^b^	not serious	not serious	not serious	none	783	799	—	MD 2.73 SD lower (3.81 lower to 1.66 lower)	⊕⊕⊕○ Moderate	
NIHSS - 30 days to 90 days												
2	randomized trials	serious^c^	not serious	not serious	not serious	none	157	163	—	MD 2.82 SD lower (6.04 lower to 0.41 higher)	⊕⊕⊕○ Moderate	
Barthel index												
2	randomized trials	serious	not serious	not serious	not serious	none	149	155	—	MD 5.95 higher (3.92 higher to 7.98 higher)	⊕⊕⊕○ Moderate	
The volume of hematoma 7 days												
3	randomized trials	serious^d^	not serious	not serious	not serious	none	152	158	—	MD 2.34 lower (3.99 lower to 0.69 lower)	⊕⊕⊕○ Moderate	
The volume of hematoma 14 days												
4	randomized trials	serious^e^	not serious	not serious	not serious	none	122	122	—	MD 2.53 lower (3.66 lower to 1.41 lower)	⊕⊕⊕○ Moderate	
The volume of hematoma 56 days												
2	randomized trials	serious^f^	not serious	not serious	not serious	none	80	80	—	MD 2.54 lower (3.34 lower to 1.75 lower)	⊕⊕⊕○ Moderate	
The volume of cerebral edema 7 days												
2	randomized trials	serious^g^	not serious	not serious	not serious	none	83	83	—	MD 2.57 lower (3.21 lower to 1.93 lower)	⊕⊕⊕○ Moderate	
The volume of cerebral edema 14 days												
4	randomized trials	serious^h^	not serious	not serious	not serious	none	182	182	—	MD 3.8 lower (4.21 lower to 3.39 lower)	⊕⊕⊕○ Moderate	

CI, confidence interval; MD, mean difference; RR, risk ratio.

a One RCT did not conduct the method of correct randomization, and five RCTs did not conduct allocation concealment. Only one RCT conducted double-blindness, and the rest of included RCTs did not conduct double-blindness. All RCTs have the risk of bias in the measurement of outcomes.

^b^One RCT did not conduct the method of correct randomization, eight RCTs did not conduct allocation concealment, nine did not conduct double-blindness, and have the risk of bias in measuring outcomes.

c One RCTs did not conduct allocation concealment and double-blindness and have the risk of bias in measuring outcomes.

d Two RCTs did not conduct allocation concealment and have the risk of bias in measuring outcomes.

e Three RCTs did not conduct allocation concealment, and two RCTs did not conduct double-blindness. All RCTs have the risk of bias in the measurement of outcomes.

f Two RCTs did not conduct allocation concealment, one RCTs do not conduct double-blindness. All RCTs have the risk of bias in the measurement of outcomes.

g Two RCTs did not conduct allocation concealment and double-blindness and have the risk of bias in measuring outcomes.

h Three RCTs did not conduct allocation concealment and double-blindness. Four RCTs did not conduct double-blindness and have the risk of bias in measuring outcomes.

## Discussion

### Summary of findings

Increasing evidence indicates that BACM plays a role in neuroprotection and inhibition of inflammatory responses and has the potential to be used in hemorrhagic stroke treatment. In this meta-analysis, we evaluated 15 RCTs, and the result showed that BACM combined with WMT was more effective than WTM alone. These positive effects are mainly consistent with the systematic review of BACM combined with WMT in ICH treatment due to their efficacy in activating blood circulation and resolving blood stasis (Xu et al., 2022).

The pathophysiologic mechanisms of post-ICH injury are complex and encompass oxidative stress, inflammation, neurotoxicity, and thrombin formation, leading to brain edema (Shao et al., 2019). In addition, peri-hematomal edema (PHE) within the first 72 h after hemorrhage leads to tissue shifts, brain herniation, and risks of clinical deterioration (Leasure et al., 2016). Conservative medical treatments include reduction of intracranial pressure, control of blood pressure, inhibition of thrombin activation, and neuroprotective agents. Surgical treatment consists of mechanical thrombectomy, craniotomy for hematoma evacuation, minimally invasive surgery, and decompressive craniotomy (Chinese Society of Neurology and Chinese Stroke Society, 2019; Ni et al., 2020). Nowadays, practical pharmacological approaches for ICH are still lacking. In addition, surgical treatment does not improve the long-term prognosis for most patients with ICH (Ni et al., 2020).

Among the commonly used herbs for blood circulation, their efficacy in improving blood circulation in patients with ICH has been proven. Our study also showed that the herbs representing BACH were *Conioselinum anthriscoides ‘Chuanxiong’* [Apiaceae], *Camellia reticulata Lindl.* [Theaceae], *Bupleurum sibiricum var. jeholense* (Nakai) C.D.Chu [Apiaceae], *Prunus persica* (L.) *Batsch* [Rosaceae], *Rheum palmatum L.* [Polygonaceae], Earthworm, *Astragalus mongholicus Bunge* [Fabaceae], *Salvia miltiorrhiza Bunge* [Lamiaceae], *Angelica sinensis* (Oliv.) Diels [Apiaceae], *Panax notoginseng* (Burkill) F.H.Chen [Araliaceae], *Achyranthes bidentata Blume* [Amaranthaceae], Leech, *Acorus calamus L.* [Acoraceae], *Rehmannia glutinosa* (Gaertn.) DC. [Orobanchaceae], and *Gastrodia elata Blume* [Orchidaceae]. In addition, some herbs, such as *Rheum palmatum L.* [Polygonaceae], *Rehmannia glutinosa* (Gaertn.) DC. [Orobanchaceae], *Acorus calamus L.* [Acoraceae], and *Gastrodia elata Blume* [Orchidaceae] were also considered adjuvant for ICH in the acute or postoperative recovery stage in recent studies (Li et al., 2015).

### Molecular mechanisms underlying the therapeutic effect of commonly used BACM for ICH

(1) *Rheum palmatum L.* [Polygonaceae]: *Rheum palmatum L.* [Polygonaceae] is a traditional and famous Chinese medicinal herb. The expression of APQ4, tumor necrosis factor-α (TNF-α), and interleukin-1β (IL-1β) was increased in ICH, resulting in disruption of the blood-brain barrier (BBB) (Bimpis et al., 2015; Wang et al., 2015). Recently, modern pharmacological experimental studies showed that rhubarb significantly reduced levels of these inflammatory factors in the serum, alleviated a variety of inflammatory responses, and protected the BBB (Yuan et al., 2017). Moreover, a systematic review showed that rhubarb combined with WMT was proven to be efficacious in improving patients’ prognosis by accelerating the absorption of cerebral hematoma and ameliorating their neurological deficits (Liu et al., 2015). An upcoming randomized controlled clinical trial will investigate the safety and efficacy of *Rheum palmatum L.* [Polygonaceae] in managing acute ICH, which may again validate the importance of BACH in the treatment of ICH by promoting blood circulation and removing blood stasis. (ClinicalTrials. gov. https://clinicaltrials.gov/ [Accessed April 1, 2022]).
(2) *Rehmannia glutinosa* (Gaertn.) DC. [Orobanchaceae]: Catalpol, an iridoid compound extracted from *Rehmannia glutinosa* (Gaertn.) DC. [Orobanchaceae], is one of the active components of *Rehmannia glutinosa.* In animal experiments, it has been shown that *Rehmannia glutinosa* accelerated the procoagulant effects by shortening the thrombin time and prothrombin time, activated partial thromboplastin time, and reducing the content of fibrinogen, thereby modulating the intrinsic and extrinsic coagulation pathways (Jia et al., 2014; Yang, 2016). Additionally, the protective effect on the BBB was proven (Liu, 2018; Zhao et al., 2021). Furthermore, *Rehmannia glutinosa* decoction based on Rehmannia can mitigate neurological deficits and improve activities of daily living (Barthel index) for patients with ICH receiving surgery, which may be related to the increased expression of Notch1 and increased activation of Notch signaling pathway (Zhang et al., 2020; Li and Zhao, 2015).
(3) *Gastrodia elata Blume* [Orchidaceae]: it has antioxidant effects through up-regulating the PI3K signaling pathway and down-regulating the level of extracellular glutamate (Zhan et al., 2016). In animal models, it has been shown to accelerate absorption of cerebral hematoma, which is associated with up-regulating cellular immunity and proliferation of microvessels (Li and Wu, 2007). Besides, a study showed that compared with WMT alone, *Gastrodia elata Blume* [Orchidaceae] combined with WMT improved neurological functions and increased the clinical efficacy (He et al., 2012).
(4) *Acorus calamus L.* [Acoraceae]: Both volatile oil and β-asarone in *Acorus calamus L.* [Acoraceae] were the main active components (Irie and Keung, 2003). Research about *Acorus calamus L.* [Acoraceae] for ICH is uncommon, but the efficacy for cognitive dysfunction has been demonstrated (Ma et al., 2017). Two RCTs performed on patients with ICH after craniotomy have demonstrated that Changpu Yujin decoction, which consists primarily of calamus, reduces mortality and the degree of cerebral edema, relieves neurological deficits, promotes recovery, and improves prognosis (Fu et al., 2019; Li and Xiao, 2020).

At present, many trials have demonstrated the clinical efficacy of BACM (e.g: decoctions, injections, and oral forms *etc*) in the remediation of neurological deficits and improvement of prognosis. A representative is Buyang Huanwu decoction, which has been reported to significantly promote the recovery of ICH patients undergoing surgery (Jing et al., 2015; Yu et al., 2017). In addition, Xueshuantong Injection and Naoxueshu Oral Liquid also were used as an adjuvant therapy for ICH (Xu et al., 2014; Wu et al., 2017; Duan et al., 2021). Although BACM is significantly efficacious in the treatment of ICH, there is an increased risk of bleeding. It has been suggested that the risks of hematoma expansion and rebleeding must be evaluated when administering BACH to ICH patients (Ni et al., 2020). With the elucidation of the pharmacological mechanisms of herbal medicine for ICH, the mechanism of BACH will be further clarified in the future. More herbal preparations, decoctions, and injections will be considered new treatment options for patients with ICH.

## Limitations

A number of limitations were found in this meta-analysis. First, the lack of rigorous design and whether the authors were blinded in some included trials may lead to publication bias and overestimation of the efficacy of BACM for ICH. Second, the small sample size in some trials may contribute to discrepant results. Third, the included RCTs did not differentiate between TCM syndromes, resulting in inaccurate results. Fourth, varied dosages of herbs and different decoction processes increased the heterogeneity of the results. Fifth, the specific timing of adding BACM was unclear. Sixth, since all participants were from China, there is not yet sufficient evidence to prove its efficacy in other ethnic groups. Therefore, more stringent, higher quality, larger sample sized clinical trials recruiting more patients from other countries are needed to elucidate the efficacy of BACM combined with WMT for ICH.

## Implications for future research

① To clarify the design protocol and to reduce bias, studies need to register on ClinicalTrials.gov or the China Clinical Trial Registration Center before starting the trial;
② To improve research quality, it is advisable to conduct rigorous design of trials, with attention to randomization methods, allocation concealment, and blinding, according to the statement (Chan et al., 2013a; Chan et al., 2013b);
③ To conduct more high-quality clinical trials, increase the sample size, and include patients from other ethnic groups and regions;
④ To focus on “syndrome differentiation and treatment” in TCM;
⑤ Clearly record the time from the symptom onset to intake of BACM;
⑥ Extend the time of treatment and follow-up, paying attention to the long-term efficacy of BACM;
⑦ Complete the report of adverse reactions/events;
⑧ To improve the quality of the report, it is necessary to ensure that reports are complete, clear, and transparent as required (Schulz et al., 2010; Moher et al., 2012).

## Conclusion

This meta-analysis suggests that BACM in combination with WTM is superior to WMT alone in ICH treatment. BACM combined with WMT has the potential to improve the overall efficacy, Barthel’s index, accelerate the absorption of hematoma, and decrease the edema volume without increasing the incidence of adverse events or mortality. However, our conclusion is inconclusive due to the high risk of bias and substantial heterogeneity. To further validate our conclusion, more high-quality RCTs are needed (Li and Zhao, 2015; Liu et al., 2015; Wu et al., 2017; Zhang et al., 2020).

## Data Availability

The raw data supporting the conclusions of this article will be made available by the authors, without undue reservation.

## References

[B1] An b J. Kim T. J. Yoon B.-W. (2017). Epidemiology, risk factors, and clinical features of intracerebral hemorrhage: An update. J. Stroke 19 (1), 3–10. 10.5853/jos.2016.00864 28178408 PMC5307940

[B2] Bimpis A. Papalois A. Voumvourakis K. Olah O. Tiszlavicz L. Liapi C. (2015). Neuronal tumour necrosis factor-α and interleukin-1β expression in a porcine model of intracerebral haemorrhage: Modulation by U-74389G. Brain Res. 1615, 98–105. 10.1016/j.brainres.2015.04.034 25916578

[B3] Chan A. W. Tetzlaff J. M. Altman D. G. Laupacis A. Gøtzsche P. C. Krleža-Jerić K. (2013a). SPIRIT 2013 statement: Defining standard protocol items for clinical trials. Ann. Intern. Med. 158 (3), 200–207. 10.7326/0003-4819-158-3-201302050-00583 23295957 PMC5114123

[B4] Chan A. W. Tetzlaff J. M. Gotzsche P. C. Altman D. G. Mann H. Berlin J. A. (2013b). SPIRIT 2013 explanation and elaboration: Guidance for protocols of clinical trials. BMJ 346, e7586. 10.1136/bmj.e7586 23303884 PMC3541470

[B5] Chen C.-C. Chen X. Li T.-C. Lin H.-L. Chu Y.-T. Lee H.-C. (2017). PG2 for patients with acute spontaneous intracerebral hemorrhage: A double-blind, randomized, placebo-controlled study. Sci. Rep. 7 (1), 45628. 10.1038/srep45628 28361971 PMC5374535

[B6] Chen Q. W. Yao Q. Y. Zhang S. J. Lu L. M. (2021). Effect of tongqiao Huoxue decoction( 通窍活血汤) on nerve function and quality of life in patients with acute cerebral hemorrhage. Chin. Arch. Tradit. Chin. Med. 39 (05), 229–232. 10.13193/j.issn.1673-7717.2021.05.055

[B7] Chinese Society of Neurology, Chinese Stroke Society (2019). Chinese guidelines for diagnosis and treatment of acute intracerebral hemorrhage 2019. Chin. J. Neurol. (12), 994–1005. 10.5534/wjmh.2013.31.2.157

[B8] Chung J. H. Lee J. W. Jo J. K. Kim K. S. Lee S. W. (2013). A quality analysis of randomized controlled trials about erectile dysfunction. World J. Mens. Health 31 (2), 157–162. 10.5534/wjmh.2013.31.2.157 24044111 PMC3770851

[B9] Deng T. Wang Y. W Y. Y. Li B. H. Jin Y. H. Ren X. Q. (2019). Methodology for clinical practice guidelines--application of GRADEpro GDT in evidence grading of systematic reviews of interventional trial. Chin. J. Evid.-Bases Cardiovasc. Med. 11 (01), 1–5. doi: not available.

[B10] Duan J. Y. Liang X. Jia M. Du W. Q. Wang M. Lei L. (2021). Systematic review and Meta-analysis on efficacy and safety of Naoxueshu Oral Liquid in treatment of hypertensive intracerebral hemorrhage. China J. Chin. Mat. Med. 46 (12), 2984–2994. 10.19540/j.cnki.cjcmm.20210324.501 34467688

[B11] Feigin V. L. Stark B. A. Johnson C. O. Roth G. A. Bisignano C. Abady G. G. (2021). Global, regional, and national burden of stroke and its risk factors, 1990–2019: A systematic analysis for the global burden of disease study 2019. Lancet Neurol. 20 (10), 795–820. 10.1016/S1474-4422(21)00252-0 34487721 PMC8443449

[B12] Fu Q. H. Yang H. J. Xie J. Zhou B. (2019). Observation on therapeutic effect of Changpu Yujin Decoction on cerebral edema after craniotomy for acute cerebral hemorrhage. Med. J. Natl. Defending Forces Southwest China 29 (5), 611–613. 10.3969/j.issn.1004-0188.2019.05.037

[B13] Gao J. Y. Shi D. L. Gao X. L. Liu T. F. Wang L. W. Cao B. (2020). Effects of Huoxue ditan decoction combined with hyperbaric oxygen on recovery rate of neurologic function and hemodynamic level in patients with hypertensive intracerebral hemorrhage. Chin. Arch. Tradit. Chin. Med. 38 (09), 213–216. 10.13193/j.issn.1673-7717.2020.09.054

[B14] Gao L. (2016). Expert consensus on hypertensive intracerebral hemorrhage in acute stage in diagnosis and treatment combining traditional Chinese medicine and Western medicine. Chin. General Pract. 19 (30), 3641–3648. 10.3969/j.issn.1007-9572.2016.30.001

[B15] Gui Z. (2018). The clinical Observation on treating cerebral Edema after hypertensive cerebral Hemorrhage with activating Blood and water method. Master's degree. Guangzhou, China: Guangzhou University of Chinese Medicine.

[B16] He F. Xiao H. Zhang F. L. (2012). Clincal observation of gastrodin in the treatment of cerebral hemorrhage. Chin. J. Clin. Ration. Drug Use 5 (20), 12–13. 10.15887/j.cnki.13-1389/r.2012.20.117

[B17] Hemphill J. C. 3rd Greenberg S. M. Anderson C. S. Becker K. Bendok B. R. Cushman M. (2015). Guidelines for the management of spontaneous intracerebral hemorrhage: A guideline for healthcare professionals from the American heart association/American stroke association. Stroke 46 (7), 2032–2060. 10.1161/STR.0000000000000069 26022637

[B18] Higgins J. P. T. Altman D. G. Gotzsche P. C. Juni P. Moher D. Oxman A. D. (2011). The Cochrane Collaboration's tool for assessing risk of bias in randomised trials. BMJ 343, d5928. 10.1136/bmj.d5928 22008217 PMC3196245

[B19] Hostettler I. C. Seiffge D. J. Werring D. J. (2019). Intracerebral hemorrhage: An update on diagnosis and treatment. Expert Rev. Neurother. 19 (7), 679–694. 10.1080/14737175.2019.1623671 31188036

[B20] Irie Y. Keung W. M. (2003). Rhizoma acori graminei and its active principles protect PC-12 cells from the toxic effect of amyloid-beta peptide. Brain Res. 963 (1-2), 282–289. 10.1016/s0006-8993(02)04050-7 12560134

[B21] Jia X. M. Zhang Z. L. Wu R. H. (2014). Effects of fresh rehmanniae radix and its processed products on blood of fevered and bleeding rats. Chin. J. Exp. Tradit. Med. Formulae 20 (6), 127–132. 10.11653/syfj2014060127

[B22] Jing Z. J. Li Y. L. Yang J. Q. Liu Y. N. Yang W. Y. Zhou R. T. (2015). Observation on the curative effect of early application of Buyang Huanwu Decoction after hypertensive cerebral hemorrhage. Mod. J. Integr. Tradit. Chin. West. Med. 24 (31), 3463–3465. 10.3969/j.issn.1008-8849.2015.31.015

[B23] Kong W. Yang J. C. Zhang P. Y. Geng S. L. (2015). Clinical effect of Xiaozhonghuayu decoction on hypertensive cerebral hemorrhage. Pharmacol. Clin. Chin. Mat. Med. 31 (01), 311–313. 10.13412/j.cnki.zyyl.2015.01.127

[B24] Leasure A. Kimberly W. T. Sansing L. H. Kahle K. T. Kronenberg G. Kunte H. (2016). Treatment of edema associated with intracerebral hemorrhage. Curr. Treat. Options Neurol. 18 (2), 9. 10.1007/s11940-015-0392-z 26874842

[B25] Li H. Q. Wei J. J. Xia W. Li J. H. Liu A. J. Yin S. B. (2015). Promoting blood circulation for removing blood stasis therapy for acute intracerebral hemorrhage: A systematic review and meta-analysis. Acta Pharmacol. Sin. 36 (6), 659–675. 10.1038/aps.2014.139 25960132 PMC4594185

[B26] Li J.-y. Yuan L.-x. Zhang G.-m. Zhou L. Gao Y. Li Q.-b. (2016a). Activating blood circulation to remove stasis treatment of hypertensive intracerebral hemorrhage: A multi-center prospective randomized open-label blinded-endpoint trial. Chin. J. Integr. Med. 22 (5), 328–334. 10.1007/s11655-016-2467-7 27338955

[B27] Li W. W. Chen L. Liu H. C. (2016b). Research progress on the pharmacological effects of safflower yellow pigment on cerebral vascular disease. World Latest Med. Inf. 16 (A0), 71–72+78. doi: not available.

[B28] Li B. Huang Y. X. Huang Z. Q. Lin L. Z. Liang Z. (2022). Breaking blood expelling stasis method accelerates hematoma resolution after hyperacute intracerebral hemorrhage and its potential mechanism. J. Chin. Physician 24 (01), 53–58. 10.3760/cma.j.cn431274-20201026-01449

[B29] Li J. Wu S. (2007). Therapeutical effect of Salvia miltiorrhiza and gastrodin to rats after cerebral hemorrhage. J. Guizhou Med. Univ. 32 (2), 118–122. doi: not available.

[B30] Li L. P. Zhao X. P. (2015). Interventional effect of dihuang yinzi on postoperative recovery of patients with spontaneous intracerebral hemorrhage. Chin. Med. Mod. Distance Educ. China 13 (8), 47–48. 10.3969/j.issn.1672-2779.2015.08.024

[B31] Li Z. B. Xiao M. M. (2020). Observation on the effect of Changpu Yujin decoction in the treatment of acute cerebral hemorrhage after craniotomy. J. Pract. Tradit. Chin. Intern. Med. 36 (12), 1547–1548. doi: not available.

[B32] Li L. (2015a). Influence of Huoxue ditan decoction on recovery of blood neural function in patients with hypertensive cerebral hemorrhage disease. Chin. J. Exp. Tradit. Med. Formulae 21 (04), 189–192. 10.13422/j.cnki.syfjx.2015040189

[B33] Li S. J. (2015b). 52 cases of hemorrhagic apoplexy treated by activating blood and removing stasis. Henan Tradit. Chin. Med. 35 (11), 2644–2646. 10.16367/j.issn.1003-5028.2015.11.1135

[B34] Li Z. W. (2015c). Clinical observation on CAIR capsule in the treatment of hydrocephalus at acute stage after cerebral hemorrhagic stroke. Master's degree. Guangzhou, China: Guangzhou University of Chinese Medicine.

[B35] Lin X. W. Gu Y. Wang N. Wang J. Y. Liu D. L. (2022). Therapeutic effect of xifeng Huayu tongluo prescription for acute cerebral hemorrhage patients with wind-phlegm-stasis obstruction syndrome and its influence on inflammatory factors, neural factors and blood-brain barrier. J. Guangzhou Univ. Tradit. Chin. Med. 39 (04), 756–763. 10.13359/j.cnki.gzxbtcm.2022.04.005

[B36] Liu C. Y. (2018). Catalpol provides a protective effect on fibrillary Aβ1-42-induced barrier disruption in an in viro model of the blood-brain barrier. Master's degree. Nanjing, China: Nanjing University Of Chinese Medicine. 10.1002/ptr.6043 29479743

[B37] Liu X. J. Zhang Y. B. Li J. M. (2015). Traditional Chinese drugs for acute intracerebral hemorrhage: A meta-analysis of randomized controlled trials. Int. J. Tradit. Chin. Med. 37 (2), 169–177. 10.3760/cma.j.issn.1673-4246.2015.02.019

[B38] Liu X. Y. Bai L. T. Ji P. H. Zhong P. F. Wang N. Y. Ji H. R. (2017). Research progress of hirudin. Asia-Pac. Tradit. Med. 13 (16), 50–52. doi: not available.

[B39] Liu Y. Zhang N. Ai M. (2019). Clinical effect of the method of promoting blood circulation and removing blood stasis in the treatment of hypertensive cerebral hemorrhage. Chin. J. Gerontology 39 (11), 2605–2607. 10.3969/j.issn.1005-9202.2019.11.011

[B40] Liu Y. Z. Guo W. F. (2009). Research on the origin of the theory of stroke pathogenesis. Chin. J. Integr. Med. Cardiovasc. Cerebrovasc. Dis. 7 (6), 732–733. 10.3969/j.issn.1672-1349.2009.06.055

[B41] Lv Y. Yu J. Yu X. Wu C. H. Li Z. M. (2021). Effect of sanyu tongluo decoction( 散瘀通络汤) on IL - 17 and related factors of NF - κBp65 pathway in patients with hypertensive cerebral hemorrhage. Chin. Arch. Tradit. Chin. Med. 39 (07), 200–204. 10.13193/j.issn.1673-7717.2021.07.050

[B42] Ma Q. R. Sun J. P. Ma J. B. Liu G. H. Liang l. Zhao J. (2013). Effects of safflower injection at different time points on cerebral hemorrhage in rats with peripheral nerve cells Caspase-3. China J. Pharm. Econ. (S3), 29–30. doi: not available.

[B43] Ma Y. X. Li G. H. Liu J. Fan Y. B. Huang Y. L. Yang D. H. (2017). Effects of β-asarone on synaptic plasticity of hippocampal neurons in Alzheimer's disease rats. Guangdong Med. J. 38 (10), 1489–1492. 10.13820/j.cnki.gdyx.20170505.022

[B44] Moher D. Hopewell S. Schulz K. F. Montori V. Gøtzsche P. C. Devereaux P. J. (2012). CONSORT 2010 explanation and elaboration: Updated guidelines for reporting parallel group randomised trials. Int. J. Surg. 10 (1), 28–55. 10.1016/j.ijsu.2011.10.001 22036893

[B45] Ni X. J. Chen Y. L. Cai Y. F. Liao W. Luo X. Wu D. (2020). Evidence-based practice guideline on integrative medicine for stroke 2019. J. Evid. Based. Med. 20 (08), 137–152. 10.1111/jebm.12386 32445289

[B46] Page M. J. McKenzie J. E. Bossuyt P. M. Boutron I. Hoffmann T. C. Mulrow C. D. (2021). The PRISMA 2020 statement: An updated guideline for reporting systematic reviews. BMJ 372, n71. 10.1136/bmj.n71 33782057 PMC8005924

[B47] Pang J. Hou J. Zhou Z. Ren M. Mo Y. Yang G. (2020). Safflower yellow improves synaptic plasticity in APP/PS1 mice by regulating microglia activation phenotypes and BDNF/TrkB/ERK signaling pathway. Neuromolecular Med. 22 (3), 341–358. 10.1007/s12017-020-08591-6 32048142

[B48] Poon M. T. C. Fonville A. F. Al-Shahi Salman R. (2014). Long-term prognosis after intracerebral haemorrhage: Systematic review and meta-analysis. J. Neurol. Neurosurg. Psychiatry 85 (6), 660–667. 10.1136/jnnp-2013-306476 24262916

[B49] Qiu S. S. Xue L. F. Li K. X. Tan F. M. Liu B. (2017). Effects of panax notoginseng saponins on neuronal apoptosis and related genes in rats with intracerebral hemorrhage. J. Jinan Univ. Nat. Sci. Med. Ed. 38 (05), 387–392. doi: not available.

[B50] Qiu Y. Li X. B. Gu X. (2021). Clinical effects of Yiqi Huoxue Huayu Decoction combined with conventional treatment on patients with hypertensive intracerebral hemorrhage after minimally invasive surgery. Chin. Tradit. Pat. Med. 43 (12), 3339–3343. doi: not available.

[B51] Qureshi A. I. Tuhrim S. Broderick J. P. Batjer H. H. Hondo H. Hanley D. F. (2001). Spontaneous intracerebral hemorrhage. N. Engl. J. Med. 344 (19), 1450–1460. 10.1056/NEJM200105103441907 11346811

[B52] Qureshi A. I. Mendelow A. D. Hanley D. F. (2009). Intracerebral haemorrhage. Lancet 373 (9675), 1632–1644. 10.1016/S0140-6736(09)60371-8 19427958 PMC3138486

[B53] Schulz K. F. Altman D. G. Moher D. (2010). CONSORT 2010 statement: Updated guidelines for reporting parallel group randomised trials. Trials 11, 32. 10.1186/1745-6215-11-32 21350618 PMC3043330

[B54] Shao Z. Tu S. Shao A. (2019). Pathophysiological mechanisms and potential therapeutic targets in intracerebral hemorrhage. Front. Pharmacol. 10, 1079. 10.3389/fphar.2019.01079 31607923 PMC6761372

[B55] Song J. N. (2000). The relationship between phlegm and phlegm and blood stasis from the perspective of biochemistry. Chin. J. Basic Med. Traditional Chin. Med. 6 (03), 40–43. 10.3969/j.issn.1006-3250.2000.03.013

[B56] Staykov D. Huttner H. B. Köhrmann M. Bardutzky J. Schellinger P. D. (2010). Novel approaches to the treatment of intracerebral haemorrhage. Int. J. Stroke 5 (6), 457–465. 10.1111/j.1747-4949.2010.00487.x 21050402

[B57] Sun C., H. (2017). Clinical observation of Naoxueshu oral liquid in preventing and treating secondary brain injury after hypertensive intracerebral hemorrhage. Master's degree. Shanghai, China: Shanghai University of Traditional Chinese Medicine.

[B58] Sun Y. M. Lou J. T. Huang G. Q. (2008). Clinical study on sodium ferulate for intracerebral hemorrhage in early stage. China J. Chin. Mat. Med. 33 (21), 2545–2548. doi: not available. 19149269

[B59] Tang X. J. Feng Y. J. Cui Y. M. Cui J. B. (2020). Research progress on clinical application of hirudo in internal medicine. J. Pract. Tradit. Chin. Intern. Med. 34 (10), 86–89. 10.13729/j.issn.1671-7813.z20200300

[B60] The Fourth Session of the National Cerebrovascular Conference (1996). The norm of clinical neurologic deficit score (1995). Chin. J. Neurol. 29 (06), 62–64. doi: not available.

[B61] Wang Y. Huang J. Ma Y. Tang G. Liu Y. Chen X. (2015). MicroRNA-29b is a therapeutic target in cerebral ischemia associated with aquaporin 4. J. Cereb. Blood Flow. Metab. 35 (12), 1977–1984. 10.1038/jcbfm.2015.156 26126866 PMC4671118

[B62] Wang M. Z. Zhang L. Jiang W. F. Liao W. L. Dang C. J. Pan W. D. (2016a). Clinical therapeutic effect and mechanism study in the prevention and treatment of the second brain injury after hypertensive cerebral hemorrhage by benefiting Qi, removing stasis and resolving phlegm. World J. Integr. Tradit. West. Med. 11 (11), 1544–1547. 10.13935/j.cnki.sjzx.161118

[B63] Wang Y. Peng F. A. N. Xie G. U. I. Chen Z.-Q. Li H.-G. Tang T. A. O. (2016b). Rhubarb attenuates blood-brain barrier disruption via increased zonula occludens-1 expression in a rat model of intracerebral hemorrhage. Exp. Ther. Med. 12 (1), 250–256. 10.3892/etm.2016.3330 27347045 PMC4907008

[B64] Wei J. Q. Ma J. (2017). Effect of Naoxueshu oral liquid on perfusion of peripheral hematoma after hypertensive intracerebral hemorrhage. Chin. J. Integr. Med. Cardiovasc. Cerebrovasc. Dis. 15 (22), 2909–2912. doi: not available.

[B65] Wu W., B. Hu C., L. Yang Y., S. Guo F., Q. Sun H. Xiao J. (2007). Effects of hirudo extract liquor on tPA and PAI-1 in experimental intracerebral peri-hematoma tissues of Wistar rats. Chin. J. Neuromed. 6 (10), 993–997. doi: not available.

[B66] Wu L. Li Y. Wang X. Ren X. Zhu H. Sun Y. (2017). A systematic review and meta-analysis on the treatment of cerebral hemorrhage with NaoXueShu oral liquid. Biomed. Res. Int. 2017,1-11, 8542576. 10.1155/2017/8542576 28630871 PMC5467282

[B67] Xi Z. Z. Xu L. Y. Bao L. M. (2018). Study on the efficacy of Buyang Huanwu Decoction in the treatment of cerebral hemorrhage and its effect on IL-6, hs-CRP, TNF-α, CD62P and CD42b. Shaanxi J. Tradit. Chin. Med. 39 (12), 1674–1676. doi: not available.

[B68] Xie J. Gao H. C. Zheng X. (2016). Effect of astragaloside IV on neurological function in acute cerebral hemorrhage rats. Chin. J. Pathophysiol. 32 (10), 1905–1908. doi: not available.

[B69] Xu Q. T. (2018). Analysis of the effect of self-made Bushen Ditan Huayu Decoction in the treatment of hypertensive cerebral hemorrhage. Shenzhen J. Integr. Tradit. Chin. West. Med. 28 (05), 77–79. 10.16458/j.cnki.1007-0893.2018.05.035

[B70] Xu D. Huang P. Yu Z. Xing D. H. Ouyang S. Xing G. (2014). Efficacy and safety of panax notoginseng saponin therapy for acute intracerebral hemorrhage, meta-analysis, and mini review of potential mechanisms of action. Front. Neurol. 5, 274. 10.3389/fneur.2014.00274 25620952 PMC4288044

[B71] Xu Y. Fang R. Zhou Y. Peng X. W. Jiang Q. L. Tang J. (2022). A meta-analysis of randomized controlled trials of traditional Chinese medicine for removing blood stasis and dredging orifices in the treatment of hypertensive cerebral hemorrhage. Mod. Tradit. Chin. Med. Materia Medica-World Sci. Technol. 23 (12), 4389–4397. 10.11842/wst.20210602007

[B72] Yang B. C. (2016). A comparative study on the dynamic change law of catalpol in different time and decoction liquid during the decoction of Rehmannia glutinosa and its effect on hemostasis. China Health Stand. Manag. 7 (10), 134–136. 10.3969/j.issn.1674-9316.2016.10.095

[B73] Yang F. Yan Y. M. (2014). A brief discussion on stroke and phlegm stasis. Heilongjiang J. Traditional Chin. Med. 43 (03), 2–3. doi: not available.

[B74] Yang L. Huang Y. Cai Y. F. Du B. X. Chen H. X. Lu M. (2004). Analysis on the distribution and evolution law of phlegm and blood stasis syndrome in 1418 stroke patients. Liaoning J. Traditional Chin. Med. 31 (06), 459–460. 10.13192/j.ljtcm.2004.06.19.yangl.012

[B75] Yu C. X. Sun S. A. Zhu Y. J. Zhang H. J. (2017). Effect of Buyang Huanwu Decoction combined with low-dose mannitol on patients with cerebral edema after hypertensive intracerebral hemorrhage. Mod. J. Integr. Tradit. Chin. West. Med. 26 (16), 1789–1791. 10.3969/j.issn.1008-8849.2017.16.028

[B76] Yuan L. X. Chen C. Zhang G. M. Zhou L. Chen Y. Cui Y. F. (2015). Clinical observation on 114 cases of cerebral hemorrhage treated by activating blood and removing stasis. Hebei J. Tradit. Chin. Med. 37 (03), 357–359. doi: not available.

[B77] Yuan M. G. Li J. X. Gu H. Guo W. F. (2017). Protective effect and related mechanisms of rhubarb in intracerebral hemorrhage. Chin. Arch. Tradit. Chin. Med. 35 (7), 1766–1768. 10.13193/j.issn.1673-7717.2017.07.035

[B78] Zeng Z. Y. Wu H. J. Yang H. Zhang B. (2022). Research progress of the theory of promoting blood circulation and removing blood stasis in the treatment of hemorrhagic stroke. J. Guizhaou Univ. Traditional Chin. Med. 44 (01), 69–73. 10.16588/j.cnki.issn2096-8426.2022.01.015

[B79] Zhan H. D. Zhou H. Y. Sui Y. P. Du X. L. Wang W. H. Dai L. (2016). The rhizome of Gastrodia elata Blume - an ethnopharmacological review. J. Ethnopharmacol. 189, 361–385. 10.1016/j.jep.2016.06.057 27377337

[B80] Zhang Z. Z. Zhang B. H. Chen M. (2003). Experimental study of Danshen injection in preventing and treating cerebral edema in acute stage of cerebral hemorrhage. Zhejiang J. Integr. Tradit. Chin. West. Med. 13 (06), 29–31. doi: not available.

[B81] Zhang F. Li W. J. Hou T. T. (2017). Influence of activating blood to remove stasis and refreshment decoction for plasma MMP - 9 and neural function of cerebral hemorrhage patients with microinvasive evacuation of hematoma. Chin. Arch. Tradit. Chin. Med. 35 (05), 1254–1256. 10.13193/j.issn.1673-7717.2017.05.055

[B82] Zhang Y. Xu K. Hou W. Wei X. W. Zhang Z. Chang T. (2020). Effects of Dihuang Yinzi on regeneration of endogenous neural stem cells and expression of Notch1 protein in rats with intracerebral hemorrhage. China J. Tradit. Chin. Med. Pharm. 35 (6), 3105–3108. doi: not available.

[B83] Zhang K. C. (2018). Observation on the clinical curative effect of self-made Suihaihuayu Decoction in the treatment of hypertensive intracerebral hemorrhage(HICH). Master's degree. Haerbin, China: Heilongjiang University of Chinese Medicine.

[B84] Zhang L. T. Zhang G. M. (2021). Study on distribution of TCM syndrome elements in hemorrhagic stroke patients. Jilin J. Tradit. Chin. Med. 41 (02), 205–208. 10.13463/j.cnki.jlzyy.2021.02.020

[B85] Zhang X. Y. Xing Y. D. (2019). Theory of etiology and pathogenesis of apoplexy and its hierarchy. Shandong J. Tradit. Chin. Med. 38 (05), 418–421. 10.16295/j.cnki.0257-358x.2019.05.005

[B86] Zhao D. Lu Y. Yu G. (2021). Effects of on behavior and blood-brain barrier in Alzheimer's disease mice. J. Zhejiang Univ. Med. Sci. 50 (5), 553–560. 10.3724/zdxbyxb-2021-0056 PMC8732245 34986530

[B87] Zhu H. M. Yu J. X. Wang N. Wang L. J. Che Y. F. (2019). Recent advance in protective mechanism of regulatory T cells in secondary injury of intracerebral hemorrhage. Chin. J. Neuromedicine 18 (05), 501–506. 10.3760/cma.j.issn.1671-8925.2019.05.012

