# Supplementary material for: Efficacy and safety of traditional Chinese medicine for intracranial hemorrhage by promoting blood circulation and removing blood stasis: A systematic review and meta-analysis of randomized controlled trials
Source: Front Pharmacol. 2022 Sep 28;13:942657. doi: 10.3389/fphar.2022.942657 (PMC9553997; doi:10.3389/fphar.2022.942657)
Supplement: Supplementary file 2 [file DataSheet1.docx]

Supplementary Material

# Supplementary text

For retrieval of databases, the keywords of search are the same, with taking pubmed as an example: (((((multi-center prospective[Title/Abstract]) OR (blinding[Title/Abstract])) OR (Parallel control experiment[Title/Abstract])) OR ("Randomized Controlled Trial" [Publication Type] OR "Randomized Controlled Trials as Topic"[Mesh])) AND (("Intracranial Hemorrhages"[Mesh]) OR ("Cerebral Hemorrhage"[Mesh]))) AND (((((SHU XUE[Title/Abstract]) OR (HUA YU[Title/Abstract])) OR (HUO XUE[Title/Abstract])) OR (blood act stasis remove agents[Title/Abstract])) OR (ACTIVATING BLOOD[Title/Abstract])).

# Supplementary Figures and Tables

## Supplementary Figures


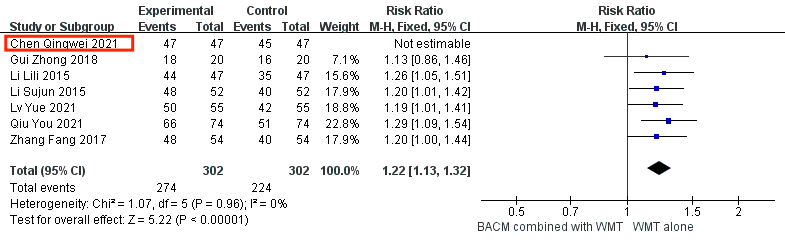


**Supplementary Figures 1** Sensitivity analysis of incidence of clinical efficacy by using BACM for ICH


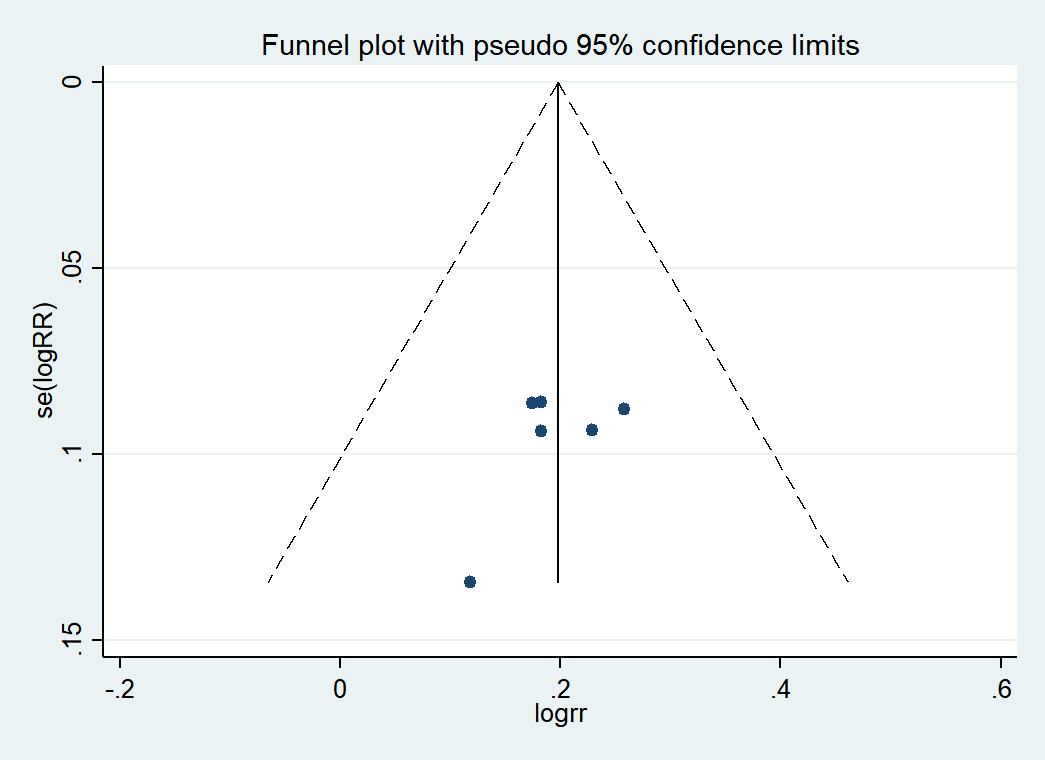


## Supplementary Figures 2 The forest plot of incidence of clinical efficacy by using BACM for ICH.


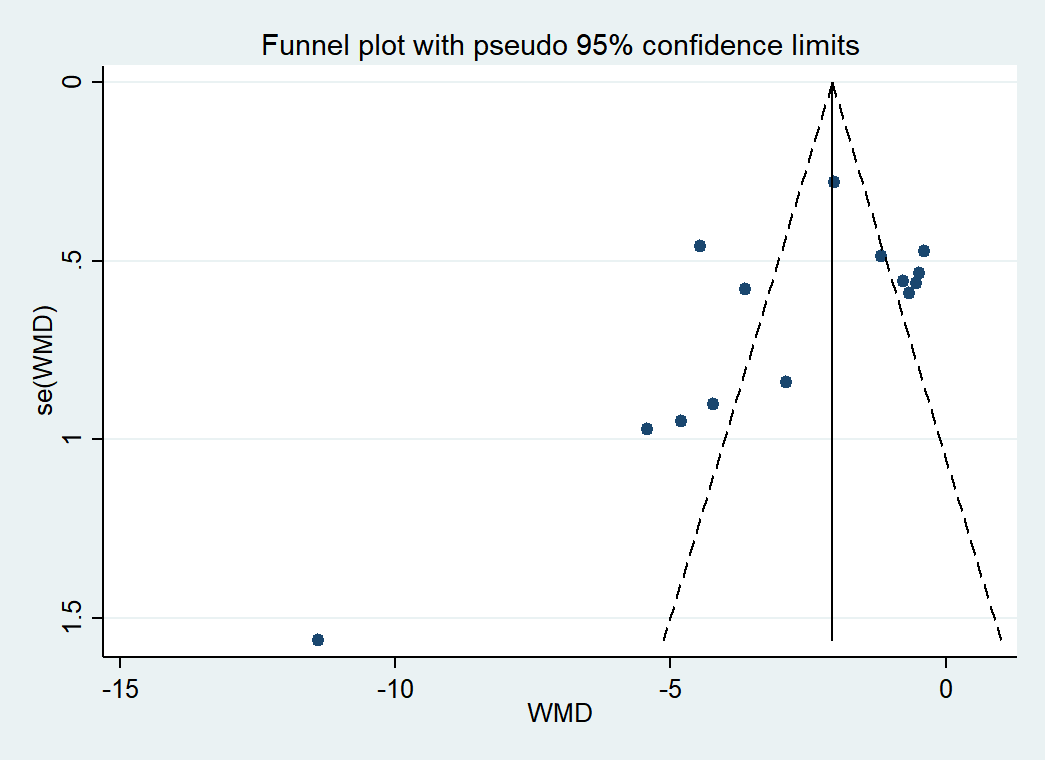


**Supplementary Figures 3** The funnel plot of improving NIHSS by using BACM for ICH


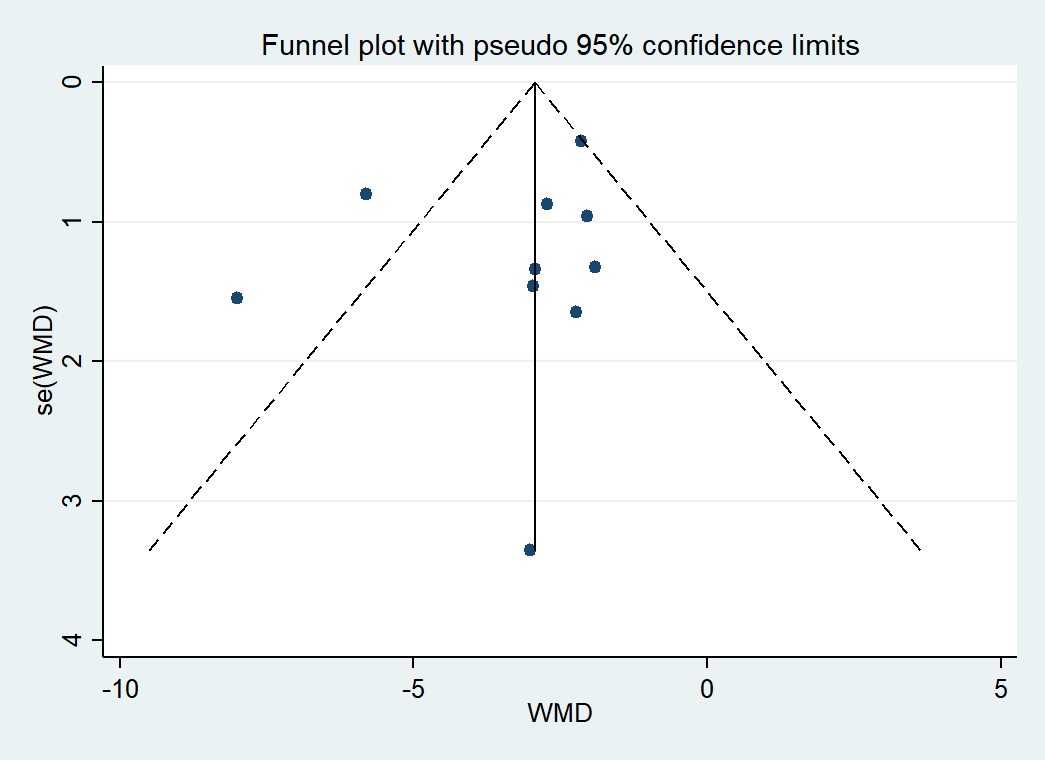


## Supplementary Figures 4 The funnel plot of volume of absorbing cerebral hematoma by using BACM for ICH

##
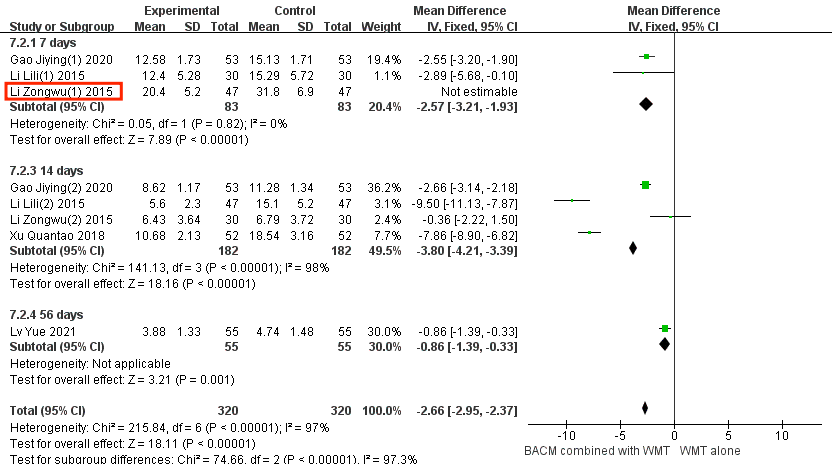


## Supplementary Figures 5 Sensitivity analysis of cerebral edema of using BACM for ICH


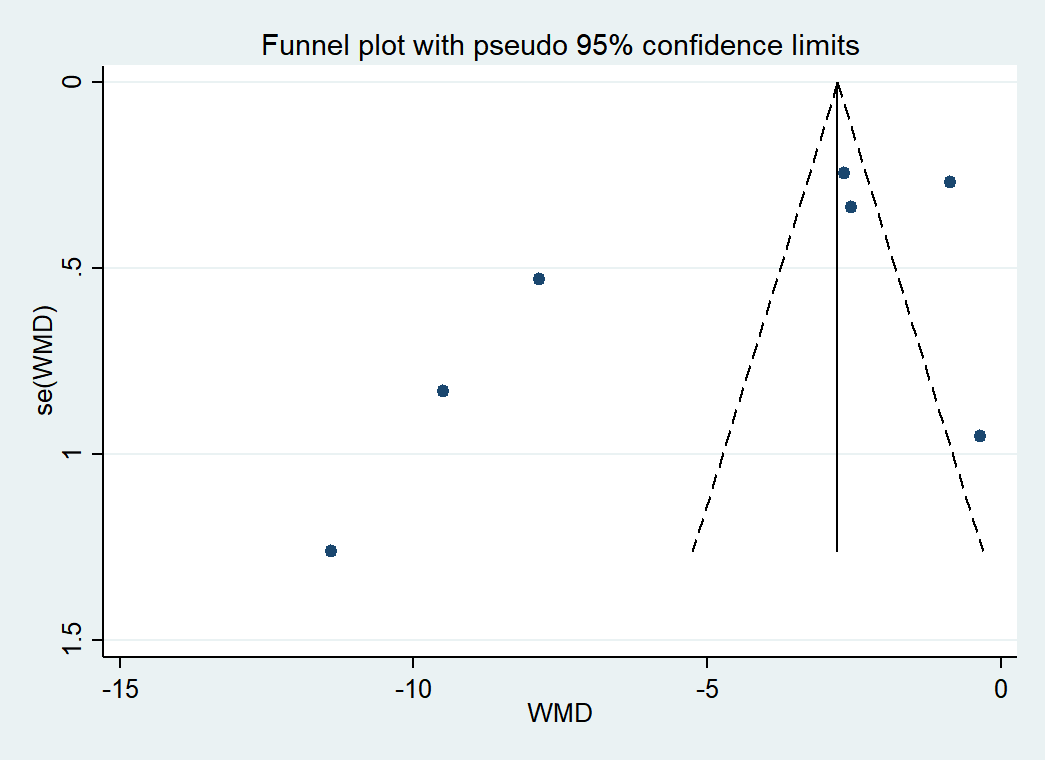


## Supplementary Figures 6 The funnel plot of cerebral edema of using BACM for ICH

## Supplementary Tables

| **Zhang 2018** | | |
| --- | --- | --- |
| **Study characteristics** | | |
| Methods | Randomised, controlled trial, single-blind | |
|  | Exclusions during trial: 0 | |
| Participants | China | |
|  | 40 participants | |
|  | 57.5% male | |
|  | Blood-activating Chinese medicine: 20; 12 male, 8 female; mean age 62.20 (SD 9.07) years; basal nuclear region 16; lobe 1; pons 1; cerebellum 1; ventricle 1; 15 hot card type; 5 non-heat type | |
|  | Western medicine treatment: 20; 11 male, 9 female; age 61.05 (SD 6.89) years; basal nuclear region 14; lobe 2; pons 1; cerebellum 2; ventricle 1; 13 hot card type; 7 non-heat type | |
|  | Intracranial hemorrhage ≤ 24 hours since time onset | |
| Interventions | Treatment: Self-made Suihai Huayu Decoction + control group treatment | |
|  | Control: Mannitol Injection + Sodium nitroprusside injection + Spearhead viper hemocoagulase injection | |
|  | Duration: 2 weeks | |
| Outcomes | Clinical efficacy | |
| Notes | Follow-up: 1 and 2 weeks | |
| ***Risk of bias*** | | |
| **Bias** | **Authors' judgement** | **Support for judgement** |
| Random sequence generation (selection bias) | Low risk | Random number sequence conducted by research team based on random number table |
| Allocation concealment  (selection bias) | high risk | Random number table used for allocation concealment |
| Blinding of participants  and personnel (performance bias)  all outcomes | unclear | Conducting single-blind approach |
| Blinding of outcome assessment (detection bias)  all outcomes | high risk | NO blinding methods used for results analyst |
| Incomplete outcome data  (attrition bias)  all outcomes | Low risk | No missing outcome data |
| Selective reporting (reporting bias) | Low risk | Study protocol not found but expected outcomes included and prespecified. |
| Other bias | Low risk | No other sources of bias identified |

**Supplementary Table 1**  Zhang’s study characteristics (Zhang, 2018 ).

| **Chen 2021** | | |
| --- | --- | --- |
| **Study characteristics** | | |
| Methods | Randomised, controlled trial | |
|  | Exclusions during trial: 0 | |
| Participants | China | |
|  | 94 participants | |
|  | 68.01% male | |
|  | Blood-activating Chinese medicine: 47; 30 male; 17 female; mean age 54.68 (SD 5.27) years; basal nuclear region 22; thalamus 5; brain stem 7; cerebellum 13; | |
|  | Western medicine treatment: 47; 34 male; 13 female; mean age 55.38 (SD 6.41) years; basal nuclear region 18; thalamus 8; brain stem 9; cerebellum 12; | |
|  | Intracranial hemorrhage ＜ 24 hours since time onset | |
| Interventions | Treatment: Tongqiao Huoxue Decoction + control group treatment | |
|  | Control: Fasudil hydrochloride injection + Hemagglutinin injection + Mannitol injection + Dexmedetomidine hydrochloride injection + Naloxone hydrochloride injection | |
|  | Duration: 4 weeks | |
| Outcomes | Clinical efficacy, NIHSS, Barthel Index and Side effects | |
| Notes | Follow-up: 4 weeks | |
| ***Risk of bias*** | | |
| **Bias** | **Authors' judgement** | **Support for judgement** |
| Random sequence generation (selection bias) | high risk | Random number sequence conducted by research team based on predetermined birthday date |
| Allocation concealment  (selection bias) | high risk | Groups divided by research team based on predetermined birthday date |
| Blinding of participants  and personnel (performance bias)  all outcomes | Low risk | No concerns in blinding of outcome assessment. |
| Blinding of outcome assessment (detection bias)  all outcomes | high risk | No blinding methods used for results analyst |
| Incomplete outcome data  (attrition bias)  all outcomes | Low risk | No missing outcome data |
| Selective reporting (reporting bias) | Low risk | Study protocol not found but expected outcomes included and prespecified. |
| Other bias | Low risk | No other sources of bias identified |

**Supplementary Table 2** Chen’s study characteristics (Chen et al., 2021).

| **Gao 2020** | | |
| --- | --- | --- |
| **Study characteristics** | | |
| Methods | Randomised, controlled trial | |
|  | Exclusions during trial: 0 | |
| Participants | China | |
|  | 106 participants | |
|  | 56.60% male | |
|  | Blood-activating Chinese medicine: 53; 29 male; 24 female; mean age 42.57 (SD 10.54) years | |
|  | Western medicine treatment: 53; 31 male; 22 female; mean age 43.02 (SD 11.04) years | |
|  | Intracranial hemorrhage ＜ 72 hours since time onset | |
| Interventions | Treatment: Huoxue Ditan Decoction + control group treatment | |
|  | Control: Routine therapy of reducing intracranial pressure + Hyperbaric oxygen therapy + Symptomatic treatment | |
|  | Duration: 2 weeks | |
| Outcomes | Clinical efficacy and The volume of cerebral edema | |
| Notes | Follow-up: 1 and 2 weeks | |
| ***Risk of bias*** | | |
| **Bias** | **Authors' judgement** | **Support for judgement** |
| Random sequence generation (selection bias) | low risk | Random number sequence conducted by research team based on random number table |
| Allocation concealment  (selection bias) | high risk | Random number table used for allocation concealment |
| Blinding of participants  and personnel (performance bias)  all outcomes | high risk | No blinding methods used for authors and participants |
| Blinding of outcome assessment (detection bias)  all outcomes | high risk | No blinding methods used for results analyst |
| Incomplete outcome data  (attrition bias)  all outcomes | Low risk | No missing outcome data |
| Selective reporting (reporting bias) | Low risk | Study protocol not found but expected outcomes included and prespecified. |
| Other bias | Low risk | No other sources of bias identified |

**Supplementary Table 3**  Gao’s study characteristics (Gao et al., 2020).

| **Xu 2018** | | |
| --- | --- | --- |
| **Study characteristics** | | |
| Methods | Randomised, controlled trial | |
|  | Exclusions during trial: 0 | |
| Participants | China | |
|  | 104 participants | |
|  | 61.54% male | |
|  | Blood-activating Chinese medicine: 52; 33 male; 19 female; mean age 61.59 (SD 4.87) years | |
|  | Western medicine treatment: 52; 31 male; 21 female; mean age 62.18 (SD 5.03) years | |
|  | Intracranial hemorrhage ＜ 67 hours since time onset | |
| Interventions | Treatment: Self-made Bushen Ditan Huayu Decoction + control group treatment | |
|  | Control: Routine therapy of reducing intracranial pressure + Symptomatic treatment | |
|  | Duration: 2 weeks | |
| Outcomes | The volume of cerebral edema | |
| Notes | Follow-up: 2 weeks | |
| ***Risk of bias*** | | |
| **Bias** | **Authors' judgement** | **Support for judgement** |
| Random sequence generation (selection bias) | Low risk | Random number sequence conducted by research team based on random number table |
| Allocation concealment  (selection bias) | Low risk | Opaque envelope used for allocation concealment |
| Blinding of participants  and personnel (performance bias)  all outcomes | high risk | No blinding methods used for authors and participants |
| Blinding of outcome assessment (detection bias)  all outcomes | high risk | NO blinding methods for results analyst |
| Incomplete outcome data  (attrition bias)  all outcomes | Low risk | No missing outcome data |
| Selective reporting (reporting bias) | Low risk | Study protocol not found but expected outcomes included and prespecified. |
| Other bias | Low risk | No other sources of bias identified |

**Supplementary Table 4**  Xu’s study characteristics (Xu, 2018).

| **Gui 2018** | | |
| --- | --- | --- |
| **Study characteristics** | | |
| Methods | Randomised, controlled trial, single-blind | |
|  | Exclusions during trial: unclear | |
| Participants | China | |
|  | 40 participants | |
|  | 52.5% male | |
|  | Blood-activating Chinese medicine: 20; 11 male; 9 female; mean age 55.95 (SD 9.64) years | |
|  | Western medicine treatment: 20; 10 male; 10 female; mean age 53.25 (SD 8.97) years | |
|  | Time of onset: unclear | |
| Interventions | Treatment: Zhuling Siwu Decoction + control group treatment | |
|  | Control: Mannitol injection + Antihypertensive therapy + Symptomatic treatment | |
|  | Duration: 2 weeks | |
| Outcomes | Clinical efficacy and The volume of hematoma | |
| Notes | Follow-up: 4 days and 2 weeks | |
| ***Risk of bias*** | | |
| **Bias** | **Authors' judgement** | **Support for judgement** |
| Random sequence generation (selection bias) | Low risk | Random number sequence conducted by research team based on random number table |
| Allocation concealment  (selection bias) | Low risk | Opaque envelope used for allocation concealment |
| Blinding of participants  and personnel (performance bias)  all outcomes | unclear | Conducting single-blind approach |
| Blinding of outcome assessment (detection bias)  all outcomes | high risk | NO blinding methods for results analyst |
| Incomplete outcome data  (attrition bias)  all outcomes | Low risk | No missing outcome data |
| Selective reporting (reporting bias) | Low risk | Study protocol not found but expected outcomes included and prespecified. |
| Other bias | Low risk | No other sources of bias identified |

**Supplementary Table 5**  Gui’s study characteristics (Gui, 2018).

| **Zhang 2017** | | |
| --- | --- | --- |
| **Study characteristics** | | |
| Methods | Randomised, controlled trial | |
|  | Exclusions during trial: 0 | |
| Participants | China | |
|  | 108 participants | |
|  | 62.96% male | |
|  | Blood-activating Chinese medicine: 54; 37 male; 17 female; mean age 65.2 (SD 7.1) years | |
|  | Western medicine treatment: 54; 31 male; 23 female; mean age 63.4 (SD 6.9) years | |
|  | Intracranial hemorrhage ＜ 24 hours since time onset | |
| Interventions | Treatment: Xingnao Decoction + control group treatment | |
|  | Control: Surgical treatment + Symptomatic treatment | |
|  | Duration: 2 weeks | |
| Outcomes | Clinical efficacy, NIHSS and Mortality | |
| Notes | Follow-up: 2 days, 1 week and 2 weeks | |
| ***Risk of bias*** | | |
| **Bias** | **Authors' judgement** | **Support for judgement** |
| Random sequence generation (selection bias) | Low risk | Random number sequence conducted by research team based on random number table |
| Allocation concealment  (selection bias) | high risk | Random number table used for allocation concealment |
| Blinding of participants  and personnel (performance bias)  all outcomes | high risk | No blinding methods used for authors and participants |
| Blinding of outcome assessment (detection bias)  all outcomes | high risk | NO blinding methods for results analyst |
| Incomplete outcome data  (attrition bias)  all outcomes | Low risk | No missing outcome data |
| Selective reporting (reporting bias) | Low risk | Study protocol not found but expected outcomes included and prespecified. |
| Other bias | Low risk | No other sources of bias identified |

**Supplementary Table 6**  Zhang’s study characteristics (Zhang, 2017).

| **Li 2015** | | |
| --- | --- | --- |
| **Study characteristics** | | |
| Methods | Randomised, controlled trial | |
|  | Exclusions during trial: 0 | |
| Participants | China | |
|  | 60 participants | |
|  | 58.33% male | |
|  | Blood-activating Chinese medicine: 30; 18 male; 12 female; mean age 58.53 (SD 7.77) years | |
|  | Western medicine treatment: 30; 17 male; 13 female; mean age 56.20 (SD 8.76) years | |
|  | Intracranial hemorrhage ＜ 15 hours since time onset | |
| Interventions | Treatment: Naomai Xinshen Capsules + control group treatment | |
|  | Control: Routine therapy of reducing intracranial pressure + Symptomatic treatment | |
|  | Duration: 2 weeks | |
| Outcomes | The volume of hematoma and The volume of cerebral edema | |
| Notes | Follow-up: 1 week and 2 weeks | |
| ***Risk of bias*** | | |
| **Bias** | **Authors' judgement** | **Support for judgement** |
| Random sequence generation (selection bias) | Low risk | Random number sequence conducted by research team based on random number table |
| Allocation concealment  (selection bias) | high risk | Random number table used for allocation concealment |
| Blinding of participants  and personnel (performance bias)  all outcomes | high risk | No blinding methods used for authors and participants |
| Blinding of outcome assessment (detection bias)  all outcomes | high risk | NO blinding methods for results analyst |
| Incomplete outcome data  (attrition bias)  all outcomes | Low risk | No missing outcome data |
| Selective reporting (reporting bias) | Low risk | Study protocol not found but expected outcomes included and prespecified. |
| Other bias | Low risk | No other sources of bias identified |

**Supplementary Table 7**  Li’s study characteristics (Li, 2015).

| **Yuan 2015** | | |
| --- | --- | --- |
| **Study characteristics** | | |
| Methods | Randomised, controlled trial，multi-center, random open | |
|  | Exclusions during trial: 0 | |
| Participants | China | |
|  | 228 participants | |
|  | 61.84% male | |
|  | Blood-activating Chinese medicine: 114; 71 male; 43 female; mean age 59.27 (SD 12.10) years | |
|  | Western medicine treatment: 114; 70 male; 44 female; mean age 62.06 (SD 10.75) years | |
|  | Intracranial hemorrhage ＜ 72 hours since time onset | |
| Interventions | Treatment: Huoxue Huayu Decoction + Xingnaojing injection + Naoxueshu Oral Liquid + control group treatment | |
|  | Control: Routine therapy of reducing intracranial pressure + Symptomatic treatment | |
|  | Duration: 3 weeks | |
| Outcomes | Barthel Index | |
| Notes | Follow-up: 1 week and 3 months | |
| ***Risk of bias*** | | |
| **Bias** | **Authors' judgement** | **Support for judgement** |
| Random sequence generation (selection bias) | Low risk | Random number sequence conducted by research team based on random number table |
| Allocation concealment  (selection bias) | Low risk | Opaque envelope used for allocation concealment |
| Blinding of participants  and personnel (performance bias)  all outcomes | high risk | No blinding methods used for authors and participants |
| Blinding of outcome assessment (detection bias)  all outcomes | high risk | NO blinding methods for results analyst |
| Incomplete outcome data  (attrition bias)  all outcomes | Low risk | No missing outcome data |
| Selective reporting (reporting bias) | Low risk | Study protocol not found but expected outcomes included and prespecified. |
| Other bias | Low risk | No other sources of bias identified |

**Supplementary Table 8**  Yuan’s study characteristics (Yuan et al., 2015).

| **Li 2015** | | |
| --- | --- | --- |
| **Study characteristics** | | |
| Methods | Randomised, controlled trial | |
|  | Exclusions during trial: 0 | |
| Participants | China | |
|  | 94 participants | |
|  | 55.32% male | |
|  | Blood-activating Chinese medicine: 47; 25 male; 22 female; mean age 63.2 (SD 10.4) years; putamen 20; thalamus 7; ventricle 10; lobe 10 | |
|  | Western medicine treatment: 47; 27 male; 20 female; mean age 62.4 (SD 9.1) years; putamen 22; thalamus 8; ventricle 9; lobe 8 | |
|  | Intracranial hemorrhage ＜ 96 hours since time onset | |
| Interventions | Treatment: Huoxue Ditan Decoction + control group treatment | |
|  | Control: Surgical treatment + Routine therapy of reducing intracranial pressure+ Symptomatic treatment | |
|  | Duration: 2 weeks | |
| Outcomes | Clinical efficacy, NIHSS and The volume of cerebral edema | |
| Notes | Follow-up: 1 week and 2 weeks | |
| ***Risk of bias*** | | |
| **Bias** | **Authors' judgement** | **Support for judgement** |
| Random sequence generation (selection bias) | Low risk | Random number sequence conducted by research team based on random number table |
| Allocation concealment  (selection bias) | high risk | Random number table used for allocation concealment |
| Blinding of participants  and personnel (performance bias)  all outcomes | high risk | No blinding methods used for authors and participants |
| Blinding of outcome assessment (detection bias)  all outcomes | high risk | NO blinding methods for results analyst |
| Incomplete outcome data  (attrition bias)  all outcomes | Low risk | No missing outcome data |
| Selective reporting (reporting bias) | Low risk | Study protocol not found but expected outcomes included and prespecified. |
| Other bias | Low risk | No other sources of bias identified |

**Supplementary Table 9**  Li’s study characteristics (Li, 2015).

| **Kong 2015** | | |
| --- | --- | --- |
| **Study characteristics** | | |
| Methods | Randomised, controlled trial, double-blind | |
|  | Exclusions during trial: 0 | |
| Participants | China | |
|  | 50 participants | |
|  | 64% male | |
|  | Blood-activating Chinese medicine: 25; 15 male; 10 female; mean age 59.34 (SD 12.18) years; basal nuclear region 9; thalamus 8; lobe 8 | |
|  | Western medicine treatment: 25; 17 male; 8 female; mean age 60.34 (SD 11.68) years; basal nuclear region 9; thalamus 7; lobe 9 | |
|  | Intracranial hemorrhage ＜ 24 hours since time onset | |
| Interventions | Treatment: Xiaozhong Huayu Decoction + control group treatment | |
|  | Control: Routine therapy of reducing intracranial pressure + Symptomatic treatment | |
|  | Duration: 8 weeks | |
| Outcomes | The volume of hematoma | |
| Notes | Follow-up: 8 weeks | |
| ***Risk of bias*** | | |
| **Bias** | **Authors' judgement** | **Support for judgement** |
| Random sequence generation (selection bias) | Low risk | Random number sequence conducted by research team based on random number table |
| Allocation concealment  (selection bias) | unclear | No specific mention of random assignment |
| Blinding of participants  and personnel (performance bias)  all outcomes | Low risk | Conducting double-blind approach |
| Blinding of outcome assessment (detection bias)  all outcomes | high risk | No blinding methods for results analyst |
| Incomplete outcome data  (attrition bias)  all outcomes | Low risk | No missing outcome data |
| Selective reporting (reporting bias) | Low risk | Study protocol not found but expected outcomes included and prespecified. |
| Other bias | Low risk | No other sources of bias identified |

**Supplementary Table 10**  Kong’s study characteristics (Kong et al., 2015).

| **Lv 2021** | | |
| --- | --- | --- |
| **Study characteristics** | | |
| Methods | Randomised, controlled trial, double-blind | |
|  | Exclusions during trial: 0 | |
| Participants | China | |
|  | 110 participants | |
|  | 60.91% male | |
|  | Blood-activating Chinese medicine: 55; 32 male; 23 female; mean age 64.15 (SD 13.74) years; basal nuclear region 28; hypothalamus 27 | |
|  | Western medicine treatment: 55; 35 male; 20 female; mean age 66.42 (SD 15.06) years; basal nuclear region 24; hypothalamus 31; | |
|  | Intracranial hemorrhage ＜ 6 hours since time onset | |
| Interventions | Treatment: Tongqiao Huoxue Decoction + control group treatment | |
|  | Control: Mannitol injection + Nimodipine tablets + Levoamlodipine + Edaravone injection | |
|  | Duration: 8 weeks | |
| Outcomes | Clinical efficacy, NIHSS, The volume of hematoma and The volume of cerebral edema | |
| Notes | Follow-up: 8 weeks | |
| ***Risk of bias*** | | |
| **Bias** | **Authors' judgement** | **Support for judgement** |
| Random sequence generation (selection bias) | Low risk | Random number sequence conducted by research team based on SAS software |
| Allocation concealment  (selection bias) | high risk | Random number table used for allocation concealment |
| Blinding of participants  and personnel (performance bias)  all outcomes | high risk | No blinding methods used for authors and participants |
| Blinding of outcome assessment (detection bias)  all outcomes | high risk | No blinding methods for results analyst |
| Incomplete outcome data  (attrition bias)  all outcomes | Low risk | No missing outcome data |
| Selective reporting (reporting bias) | Low risk | Study protocol not found but expected outcomes included and prespecified. |
| Other bias | Low risk | No other sources of bias identified |

**Supplementary Table 11** Lv’s study characteristics (Lv et al., 2021).

| **Li 2015** | | |
| --- | --- | --- |
| **Study characteristics** | | |
| Methods | Randomised, controlled trial, double-blind | |
|  | Exclusions during trial: 0 | |
| Participants | China | |
|  | 104 participants | |
|  | 63.46% male | |
|  | Blood-activating Chinese medicine: 52; 34 male; 18 female; mean age 67.5 (SD 7.6) years | |
|  | Western medicine treatment: 52; 32 male; 20 female; mean age 67.9 (SD 7.8) years | |
|  | Time of onset: unclear | |
| Interventions | Treatment: Huoxue Huayu Decoction + control group treatment | |
|  | Control: Routine therapy of reducing intracranial pressure + Symptomatic treatment | |
|  | Duration: 15 days | |
| Outcomes | Clinical efficacy and The volume of hematoma | |
| Notes | Follow-up: 15 days | |
| ***Risk of bias*** | | |
| **Bias** | **Authors' judgement** | **Support for judgement** |
| Random sequence generation (selection bias) | Low risk | Random number sequence conducted by research team based on random number table |
| Allocation concealment  (selection bias) | unclear | No specific mention of random assignment |
| Blinding of participants  and personnel (performance bias)  all outcomes | Low risk | Conducting double-blind approach |
| Blinding of outcome assessment (detection bias)  all outcomes | high risk | No blinding methods for results analyst |
| Incomplete outcome data  (attrition bias)  all outcomes | Low risk | No missing outcome data |
| Selective reporting (reporting bias) | Low risk | Study protocol not found but expected outcomes included and prespecified. |
| Other bias | Low risk | No other sources of bias identified |

**Supplementary Table 12**  Li’s study characteristics (Li, 2015).

| **Sun 2017** | | |
| --- | --- | --- |
| **Study characteristics** | | |
| Methods | Randomised, controlled trial | |
|  | Exclusions during trial: 19 | |
| Participants | China | |
|  | 101 participants | |
|  | 60.40% male | |
|  | Blood-activating Chinese medicine: 51; 32 male; 19 female; mean age 59.92 (SD 16.22) years | |
|  | Western medicine treatment: 50; 29 male; 21 female; mean age 63.72 (SD 15.54) years | |
|  | Intracranial hemorrhage ＜ 24 hours since time onset | |
| Interventions | Treatment: Naoxueshu Oral Liquid + control group treatment | |
|  | Control: Routine therapy of reducing intracranial pressure + Symptomatic treatment | |
|  | Duration: 12 days | |
| Outcomes | NIHSS | |
| Notes | Follow-up: 5 (SD 2) days and 10 (SD 2) days | |
| ***Risk of bias*** | | |
| **Bias** | **Authors' judgement** | **Support for judgement** |
| Random sequence generation (selection bias) | Low risk | Random number sequence conducted by research team based on random number table |
| Allocation concealment  (selection bias) | high risk | Random number table used for allocation concealment |
| Blinding of participants  and personnel (performance bias)  all outcomes | high risk | No blinding methods used for authors and participants |
| Blinding of outcome assessment (detection bias)  all outcomes | high risk | No blinding methods for results analyst |
| Incomplete outcome data  (attrition bias)  all outcomes | Low risk | No missing outcome data |
| Selective reporting (reporting bias) | Low risk | Study protocol not found but expected outcomes included and prespecified. |
| Other bias | Low risk | No other sources of bias identified |

**Supplementary Table 13**  Sun’s study characteristics (Sun, 2017).

| **Li 2016** | | |
| --- | --- | --- |
| **Study characteristics** | | |
| Methods | Randomised, controlled trial,multi-center | |
|  | Exclusions during trial: 18 | |
| Participants | China | |
|  | 210 participants | |
|  | 68.01% male | |
|  | Blood-activating Chinese medicine: 102; 31 male; 71 female; mean age 59.2 (SD 12.1) years | |
|  | Western medicine treatment: 108; 41 male; 67 female; mean age 62.1 (SD 10.8) years | |
|  | Intracranial hemorrhage ＜ 72 hours since time onset | |
| Interventions | Treatment: Huoxue Huayu Decoction + Xingnaojing injection + Naoxueshu Oral Liquid + control group treatment | |
|  | Control: Routine therapy of reducing intracranial pressure + Symptomatic treatment | |
|  | Duration: 4 weeks | |
| Outcomes | NIHSS, The volume of hematoma, Side effects and Mortality | |
| Notes | Follow-up: 1 week, 2 weeks, 3 weeks and 3 months | |
| ***Risk of bias*** | | |
| **Bias** | **Authors' judgement** | **Support for judgement** |
| Random sequence generation (selection bias) | Low risk | Random number sequence conducted by research team based on SAS software |
| Allocation concealment  (selection bias) | Low risk | Opaque envelope used for allocation concealment |
| Blinding of participants  and personnel (performance bias)  all outcomes | Low risk | Conducting singe-blind approach |
| Blinding of outcome assessment (detection bias)  all outcomes | Low risk | Conducting singe-blind approach |
| Incomplete outcome data  (attrition bias)  all outcomes | Low risk | No missing outcome data |
| Selective reporting (reporting bias) | Low risk | Study protocol not found but expected outcomes included and prespecified. |
| Other bias | Low risk | No other sources of bias identified |

**Supplementary Table 14**  Li’s study characteristics (Li et al., 2016).

| **Qiu 2021** | | |
| --- | --- | --- |
| **Study characteristics** | | |
| Methods | | |
| Participants | Exclusions during trial: 0 | |
|  | China | |
| Interventions | 148 participants | |
|  | 70.27% male | |
|  | Blood-activating Chinese medicine: 74; 49 male; 25 female; mean age 60.1 (SD 8.4) years; basal nuclear region 51; lobe 10； ventricle 13 | |
|  | Western medicine treatment: 74; 55 male; 19 female; mean age 61.5 (SD 8.8) years; basal nuclear region 54; lobe 10； ventricle 10 | |
|  | Intracranial hemorrhage ＜ 24 hours since time onset | |
|  | Treatment: Tongqiao Huoxue Decoction + control group treatment | |
| Outcomes | Control: Surgical treatment + Routine therapy of reducing intracranial pressure+ Symptomatic treatment | |
|  | Duration: 4 weeks | |
|  | Clinical efficacy, NIHSS and The volume of hematoma | |
| Notes | Follow-up: 4 weeks | |
| Risk of bias |  | |
| ***Bias*** | ***Authors' judgement*** | ***Support for judgement*** |
| Random sequence generation (selection bias) | Low risk | Random number sequence conducted by research team based on random number table |
| Allocation concealment  (selection bias) | high risk | Random number table used for allocation concealment |
| Blinding of participants  and personnel (performance bias)  all outcomes | high risk | No blinding methods used for authors and participants |
| Blinding of outcome assessment (detection bias)  all outcomes | high risk | No blinding methods for results analyst |
| Incomplete outcome data  (attrition bias)  all outcomes | Low risk | No missing outcome data |
| Selective reporting (reporting bias) | Low risk | Study protocol not found but expected outcomes included and prespecified. |
| Other bias | Low risk | No other sources of bias identified |

**Supplementary Table 15**  Qiu’s study characteristics (Qiu et al., 2021).
